# Peptide-DNA origami as a cryoprotectant for cell preservation

**DOI:** 10.1126/sciadv.add0185

**Published:** 2022-10-28

**Authors:** Chanseok Lee, Yedam Lee, Woo Hyuk Jung, Tae-Yeon Kim, Taehwi Kim, Do-Nyun Kim, Dong June Ahn

**Affiliations:** ^1^ Institute of Advanced Machines and Design, Seoul National University, Seoul 08826, Korea.; ^2^ The w:i Interface Augmentation Center, Korea University, Seoul 02841, Korea.; ^3^ Department of Chemical and Biological Engineering, Korea University, Seoul 02841, Korea.; ^4^ Department of Mechanical Engineering, Seoul National University, Seoul 08826, Korea.; ^5^ KU-KIST Graduate School of Converging Science and Technology, Korea University, Seoul 02841, Korea.

## Abstract

Cryopreservation of cells is essential for the conservation and cold chain of bioproducts and cell-based medicines. Here, we demonstrate that self-assembled DNA origami nanostructures have a substantial ability to protect cells undergoing freeze-thaw cycles; thereby, they can be used as cryoprotectant agents, because their nanoscale morphology and ice-philicity are tailored. In particular, a single-layered DNA origami nanopatch functionalized with antifreezing threonine peptides enabled the viability of HSC-3 cells to reach 56% after 1 month of cryopreservation, surpassing dimethyl sulfoxide, which produced 38% viability. It also exhibited minimal dependence on the cryopreservation period and freezing conditions. We attribute this outcome to the fact that the peptide-functionalized DNA nanopatches exert multisite actions for the retardation of ice growth in both intra- and extracellular regions and the protection of cell membranes during cryopreservation. This discovery is expected to deepen our fundamental understanding of cell survival under freezing environment and affect current cryopreservation technologies.

## Teaser

DNA nanopatches functionalized with antifreezing peptides are highly effective in survival of cells during cryopreservation.

## INTRODUCTION

Protection of cells from cryoinjuries has been a major challenge in cryopreservation. Dimethyl sulfoxide (DMSO) is one of the most common and widely used membrane-penetrating cryoprotective agents (CPAs) that hinder the formation and growth of ice crystals (*1*, *2*). The optimal concentration of DMSO mixed with cell culture media is typically as high as 10% (v/v); however, the use of high concentrations of DMSO has been shown to produce cytotoxicity and induce adverse effects when thawed cells are infused into the patient (*3*–*5*). Therefore, DMSO is occasionally used together with other chemicals such as hydroxyethyl starch, sucrose polyvinyl pyrrolidone, dextran, and trehalose for reducing cytotoxicity (*6*). In addition, other polymers that have strong ice recrystallization inhibition (IRI) activity can be used with other CPAs; these polymers include synthetic polypeptides (*7*, *8*), poly(vinyl alcohol) (PVA) (*9*, *10*), and glycoconjugates (*11*, *12*).

Another class of cell cryoprotectants is natural antifreeze (glyco-) proteins [AF(G)Ps] found in certain polar fish (*13*–*15*), water flounder (*16*, *17*), and plants (*18*). They are effectively adsorbed onto specific ice planes, resulting in the thermal hysteresis of the solution and growth inhibition of ice seeds (*19*). Such binding characteristics of certain AF(G)Ps originate from periodically arranged peptides such as alanine (Ala) or threonine (Thr) (*20*, *21*). The IRI ability of AF(G)Ps has led researchers to exploit them as cryoprotectants; when used in combination with DMSO or glycerol, the concentration of which was lower than that typically used for cryopreservation, certain AF(G)Ps showed similar effects on cryoprotective functions. However, the addition of AF(G)Ps was not necessarily successful in improving cell viability, possibly owing to the anisotropic ice-shaping behavior and poor cellular uptake efficiency (*22*). While the investigations use ice-binding proteins (*23*, *24*) and two-dimensional materials like graphene oxide (*25*, *26*) with efficient antifreeze or ice nucleation properties, there have been no reports of an alternative CPA that can be used alone and that outperforms the cell recovery by 10% DMSO to date.

**Fig. 1.**
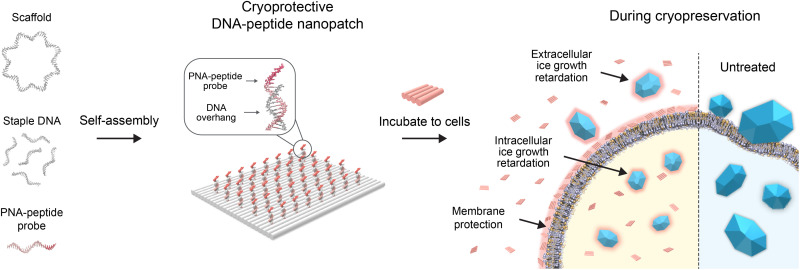
Schematic illustration of the assembly of cryoprotective DNA-peptide nanopatches and their preservation mechanisms. M13mp18 single-stranded DNA, staple DNA strands, and PNA strands decorated with antifreezing peptides were self-assembled onto DNA-peptide nanopatch structures by one-pot annealing process. After incubation with cells before freezing, they exhibited the retardation of ice growth both within extra- and intracellular domains. DNA-peptide nanopatch structures attached to the cell membrane additionally provided protection ability during the cryopreservation process.

Here, we investigated an approach to achieve higher cell viability after long-term cryopreservation (up to 1 month) using self-assembled nanoscale DNA structures. Scaffolded DNA origami nanostructures, consisting of a long single-stranded DNA and multiple short strands that fold it into a designed geometry, have been widely used as intracellular delivery carriers or membrane-attachable structures (*27*–*29*). Their intracellular uptake ability without cytotoxicity (*30*, *31*) and robust shape integrity after freeze-thaw cycles (*32*) prompted us to explore them as attractive candidates for CPA. We tested slender bundles and rectangular patch structures and found that the patch shape showed a noticeable cryoprotective performance. Inspired by the conjugation of antifreezing peptides onto gold nanoparticles (*33*), we functionalized DNA nanopatch (NP) with different types and numbers of antifreezing peptides using peptide nucleic acid (PNA)–based linkers (Fig. 1).

## RESULTS

### Design and self-assembly process

**Fig. 2.**
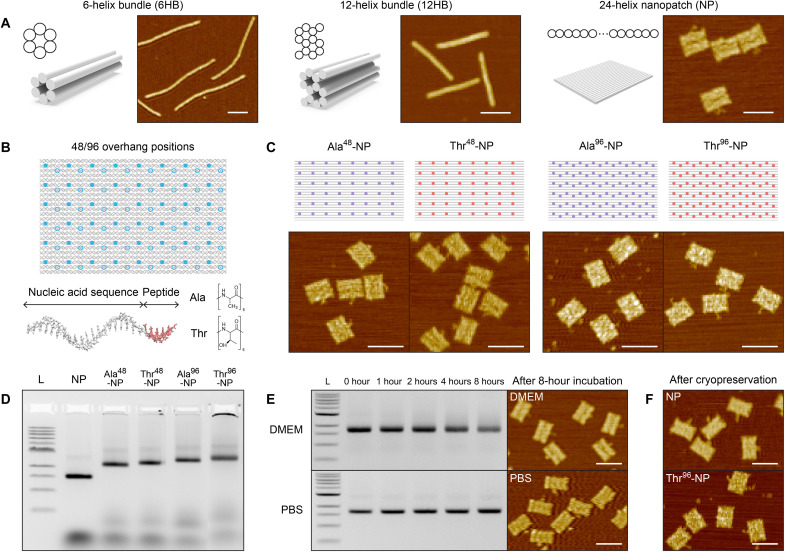
Design, self-assembly, and characterization of DNA origami–based nanostructures and DNA-peptide nanopatches. (**A**) Cross-sectional shape and representative AFM images of DNA origami bundle (6HB and 12HB) and NP structures. The detailed design and additional AFM images are presented in fig. S1. Scale bars, 100 nm. (**B**) Schematic illustration of the NP structure with possible overhang positions; the peptide-PNA probe consists of the complementary sequence with the overhang and antifreezing peptides. At the end of the PNA probe, five repeating Ala or Thr peptides are attached. (**C**) Schematic illustration and representative AFM images of the peptide-functionalized nanopatch structures. Colored dots indicate the overhang positions on NPs. Scale bars, 100 nm. (**D**) Agarose gel electrophoresis of self-assembled cryoprotective DNA origami nanostructures. Scale bars, 100 nm. (**E**) Stability analysis of NP structure, which was mixed with either DMEM (with 10% FBS) or PBS solution at a 1:9 ratio and subsequently incubated at 37°C for up to 8 hours. Scale bars, 100 nm. (**F**) AFM images of NP and Thr^96^-NP structures after 1 day of cryopreservation.

To investigate the cryoprotective performance of structured DNA assemblies, scaffolded DNA origami with lattice-packed geometry (*34*) was used to create DNA nanostructures with regular dimensions. Nanorod geometries consisting of 6 and 12 double-stranded DNA (dsDNA) helices (denoted as 6HB and 12HB, respectively) were designed with lengths of approximately 380 and 190 nm, respectively (Fig. 2A and fig. S1) (*35*). In addition, a single-layered rectangular nanopatch (90 nm by 70 nm) consisting of 24 dsDNA helices (denoted as NP) was prepared (Fig. 2A and fig. S1) (*27*). For the attachment of antifreezing peptides to the DNA nanopatch, 48 or 96 single-stranded overhangs were inserted at regular intervals, protruding at the single side of the nanopatch. We introduced a PNA-based linker consisting of two parts: (i) nucleic acid bases that are complementary to those of the overhangs and (ii) peptides of five consecutive Thr or Ala connected at its C terminus (Fig. 2B). Peptide-functionalized nanopatches were named after the peptide type and the number of overhangs such as Ala^48^-NP and Thr^96^-NP (Fig. 2C).

Programmed self-assembly of the bare and peptide-functionalized DNA origami nanostructures was performed using a conventional one-step annealing process (*36*). After assembly, excessive nucleic acid strands were purified, and MgCl_2_ concentration was reduced to 5 mM using centrifugal filtration (*37*, *38*) for enhancing the filtration yield (*36*) and for further use as a cell cryoprotectant. The DNA nanopatches with various peptide decorations were validated using agarose gel electrophoresis and atomic force microscopy (AFM) imaging (Fig. 2, C and D, and fig. S1D). To assess the stability of the DNA nanopatch structures after incubation under physiological conditions, we incubated them in Dulbecco’s modified Eagle’s medium [DMEM; 10% fetal bovine serum (FBS)] and phosphate-buffered solution (PBS) at 37°C for 8 hours. Their original shapes were maintained well, as validated using gel electrophoresis and AFM measurements (Fig. 2E). Freeze-thaw cycle in liquid nitrogen did not deteriorate the shape of bare and Thr-DNA nanopatch structures, in accordance with the previous report (*32*) and typical DNA origami storage procedure (Fig. 2F).

### Cellular uptake and cryopreservation performance

**Fig. 3.**
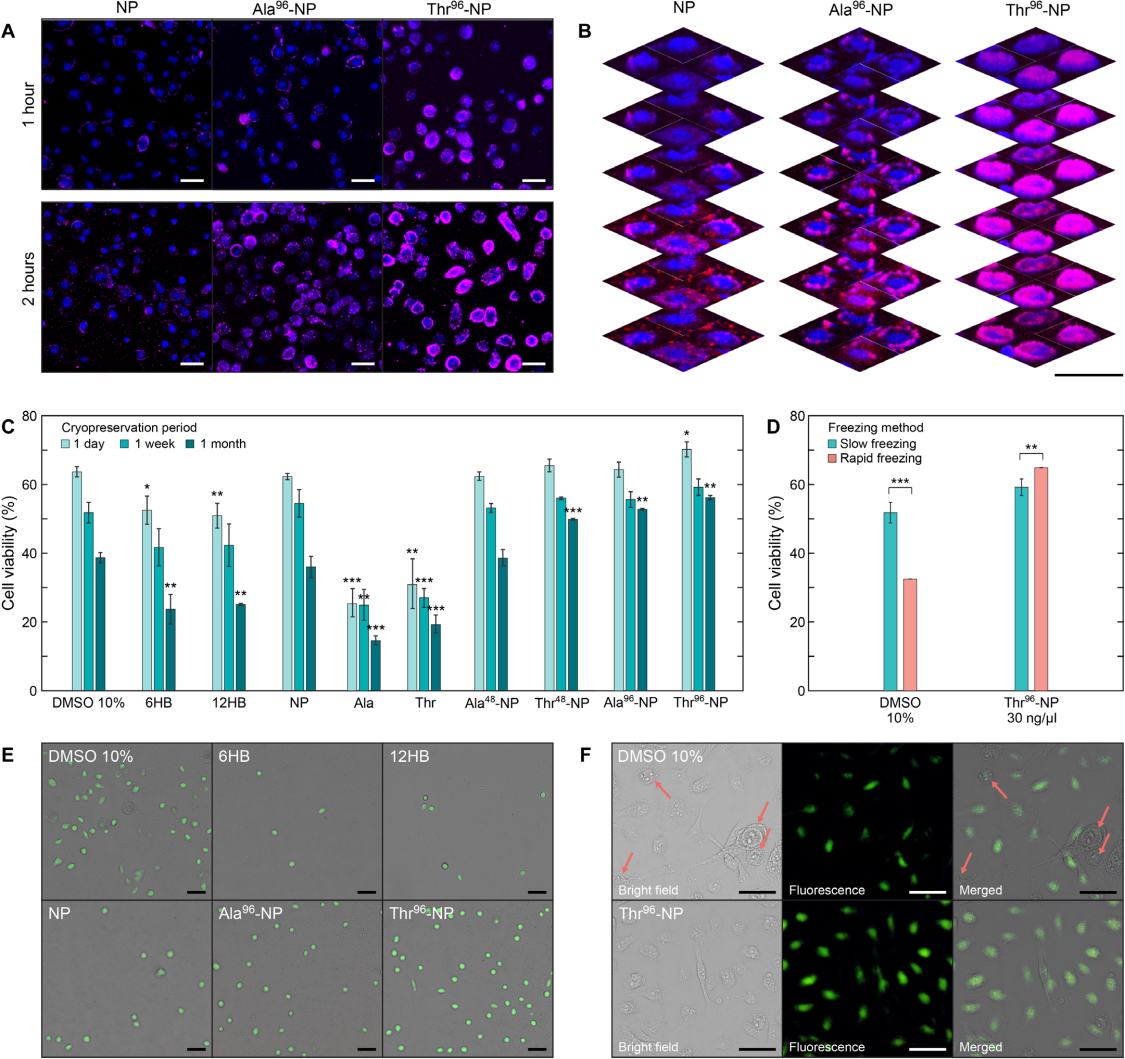
Cellular uptake and cryoprotective performance of various DNA nanopatch structures. (**A**) Confocal microscope images of HSC-3 cells incubated with the bare and peptide-functionalized NP structures (20 ng/μl) for 1 and 2 hours. Blue, Hoechst 33258 (nucleus). Magenta, Cy3 moiety on NP structures. Scale bars, 50 μm. (**B**) Representative *Z* stack confocal images of the cells incubated with NP, Ala^96^-NP, and Thr^96^-NP structures for 2 hours. Scale bar, 50 μm. (**C**) MTT assay for the cells treated with peptide-PNA probes and various cryoprotective DNA nanostructures (30 ng/μl). Cells were cryopreserved for 1 day, 1 week, and 1 month. Error bars represent SD. *P < 0.05; **P < 0.01; ***P < 0.001, compared against the values obtained with 10% DMSO for the same period of cryopreservation. All *P* values are presented in table S5. (**D**) One-week cell viability of DMSO- and Thr^96^-NP–treated cells with different freezing methods. Unlike cells incubated with DMSO, Thr^96^-NP–treated cells showed a viability slightly higher when frozen directly in the liquid nitrogen without the slow-freezing process. Error bars represent the SD. **P < 0.01; ***P < 0.001. (**E**) Bright-field and fluorescent microscope images of the postthawed cells after a 24-hour incubation on the substrate. Concentration of the DNA structure was maintained at 20 ng/μl for all cases. Green, sodium indicator. Scale bars, 50 μm. (**F**) Enlarged view of the postthawed cells treated with 10% DMSO and Thr^96^-NP structures (20 ng/μl). Red arrows indicate the ill-conditioned cells, as indicated by their morphological changes and low fluorescence intensity. Green, sodium indicator. Scale bars, 50 μm. Data in (C) and (D) are expressed as the means ± SD from three independent experiments.

When incubated with cells, DNA origami nanostructures can be attached to the cell membrane and internalized by endocytosis, while the geometry of nanostructures and cell types are known parameters for uptake efficiency (*30*, *39*). We observed the membrane attachment and intracellular uptake behavior of the bare and peptide-functionalized DNA nanopatches using confocal microscopy (Fig. 3, A and B, and fig. S2). Human oral squamous cell carcinoma (HSC-3) cells were treated with samples diluted with DMEM at a 1:9 ratio (final concentration: 20 ng/μl) and incubated for 2 hours. Cells incubated with Ala^96^-NP and Thr^96^-NP showed relatively higher fluorescence intensities than those incubated with bare DNA nanopatches. The Thr^96^-DNA nanopatch showed better membrane adhesion and intracellular uptake performance than the other nanopatches (Fig. 3B).

Next, the cryoprotective performance of the bare DNA nanostructures with different geometries (6HB, 12HB, and NP) and peptide-functionalized DNA nanopatches were assessed using 3-(4,5-dimethylthiazol-2-yl)-2,5-diphenyltetrazolium bromide (MTT) assay. Cells were incubated with various samples (30 ng/μl) for 2 hours and slowly frozen at −40°C in a cell-freezing container (Mr. Frosty) for 12 hours. The frozen cells were subsequently moved to a liquid nitrogen chamber (−196°C) and stored for 1 day, 1 week, and 1 month. After cryopreservation, the cells were rapidly warmed at 37°C, and the DMEM solutions of all samples were exchanged with PBS buffer, following which the MTT assay was performed. This process took less than 30 min after thawing. As a reference, the viability of the cells when not treated with any cryoprotectant was 17.3 ± 4.6%, and when treated with 10% DMSO, it was 63.7 ± 1.5% for 1 day of cryopreservation. 6HB, 12HB, and NP structures (30 ng/μl) showed cell viabilities of 52.5 ± 4.1%, 50.9 ± 3.6%, and 62.3 ± 0.9%, respectively, for the same period of preservation (Fig. 3C). The large area-to-mass ratio of the nanopatch design over the bundles is considered one of the reasons for the higher cryoprotective performance of the DNA nanopatch.

Note that the bare DNA nanopatch achieved high cell viability, comparable to the value achieved with 10% DMSO, even without the addition of any conventional cryoprotectants. Functionalization of the bare DNA nanopatches with antifreezing peptides could further enhance cell cryopreservation ability. Without being attached to the DNA nanopatches, Ala and Thr probes (30 ng/μl) marked as low as 25.3 ± 4.1% and 30.8 ± 7.4% of cell viability after 1 day of cryopreservation, respectively (Fig. 3C). In case of peptide-functionalized DNA nanopatches, however, the value was elevated to 62.2 ± 1.9% and 64.3 ± 2.2% using Ala^48^- and Ala^96^-NPs and 65.5 ± 3.8% and 70.2 ± 2.2% using Thr^48^- and Thr^96^-NPs, respectively. Notably, the performance of peptide-functionalized DNA nanopatches was more pronounced when the incubated cells were preserved for longer periods up to 1 month. Upon increasing the cryopreservation period, DMSO and many DNA nanostructures resulted in reduced cell viability to two-thirds to half of the basal values; however, Thr^48^-, Ala^96^-, and Thr^96^-NPs better maintained their cryoprotective performance (Fig. 3C). As the best record, Thr^96^-DNA nanopatch (30 ng/μl, ~5.3 nM) achieved 56.2 ± 0.6% after 1 month of cryopreservation and outperformed 10% DMSO by 1.5-fold. For the bare and peptide-functionalized DNA nanopatches, the effects of initial incubation time and concentration were further investigated (fig. S3). In contrast to DMSO in which incubation before freezing adversely affects the cells due to its cytotoxicity, our DNA nanopatches maintained cell viability during the initial incubation time (fig. S3A). Increasing the initial incubation time slightly increased the cell viability; however, the values tended to converge after more than 2 hours of incubation (fig. S3B). In addition, a decrease in the nanopatch sample concentration to 10 and 20 ng/μl slightly reduced the cell viability; however, the amount of reduction was up to 8.6% within the same sample type (fig. S3C).

To date, the cryoprotective performance of DNA nanostructures has been assessed under the conventional slow-freezing protocol using DMSO, which requires a mild rate of temperature change for securing the dehydration inside the cells and preventing intracellular ice formation and osmotic shock to a certain degree (*40*). However, a 1-week cryopreservation test with different freezing protocols revealed that the cryoprotection efficiency of the Thr^96^-NP (30 ng/μl) was improved when the cells were rapidly frozen in liquid nitrogen, yielding 65%, which is in stark contrast to the yield of 32% achieved with DMSO (Fig. 3D). This result implies that the underlying cryoprotective mechanism may be different between the Thr_96_-NP and DMSO, because the Thr_96_-NP exhibited higher cryopreservation ability, despite allowing intracellular ice formation. In addition to the MTT assay, the postthaw behavior of the cells cryopreserved for 1 day was assessed using fluorescence microscopy after 24 hours of additional incubation in a cell culture dish (Fig. 3E). Ala^96^- and Thr^96^-NPs showed noticeably more active cells on the substrate, similar to 10% DMSO. Certain swollen cells with a substantial loss of sodium indicator signals were observed in DMSO-treated cells, possibly indicating that their membranes were severely damaged (Fig. 3F, top row). Among the groups evaluated, the cells treated with the Thr^96^-NP generally showed better cell conditions without visible signs of swelling (Fig. 3F, bottom row).

### Retardation of extra/intracellular ice growth

**Fig. 4.**
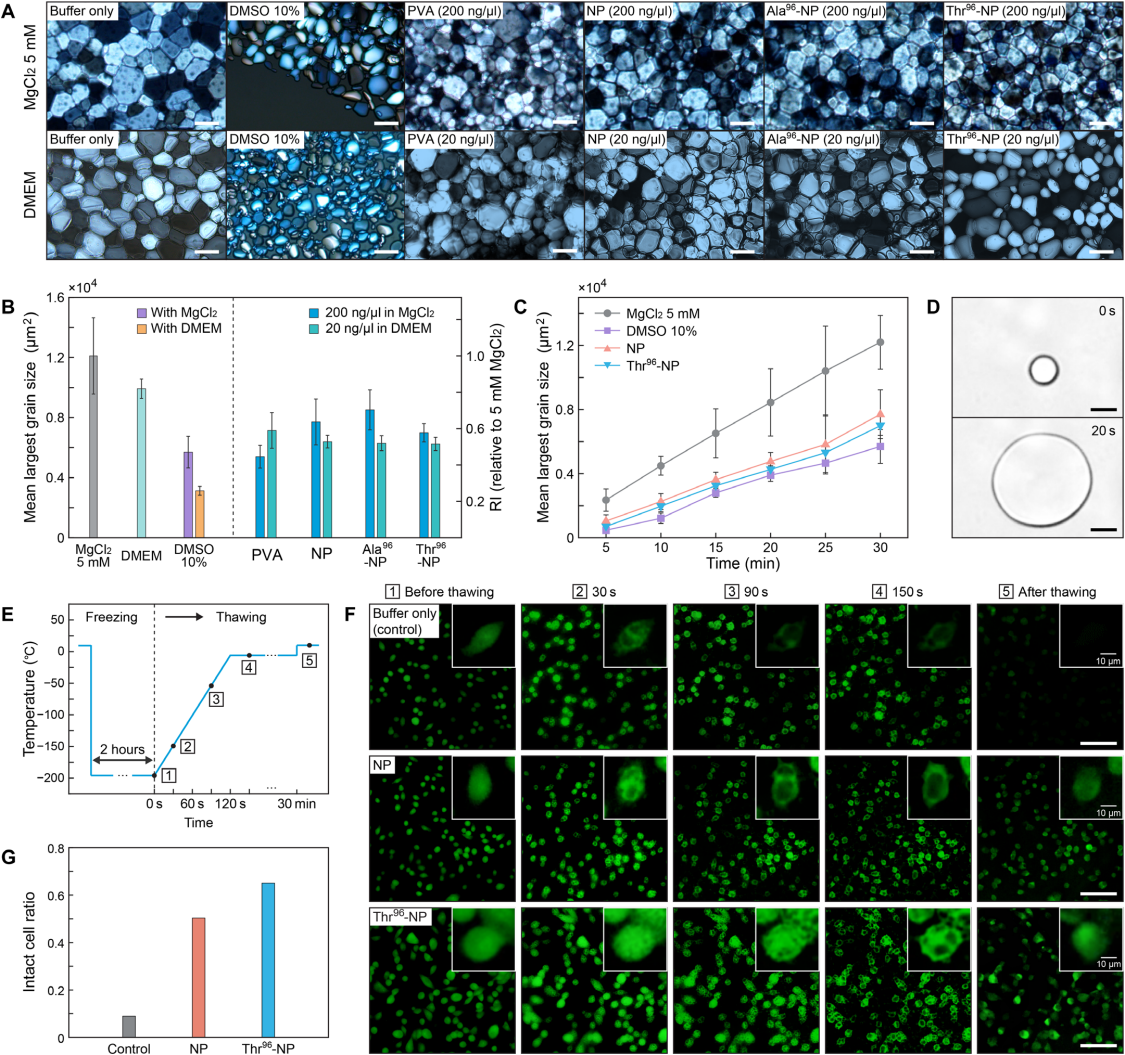
Extracellular and intracellular ice growth retardation by cryoprotective DNA nanopatches. (**A**) Polarized optical microscope images of ice grains after 30 min of incubation at −6°C. DMSO and nanopatch structures were either mixed with 5 mM MgCl_2_ or DMEM solution. Scale bars, 100 μm. (**B**) IRI result for 10% DMSO, PVA (*M*_w_: 9 kDa), NP, and peptide-functionalized nanopatch structures with 5 mM MgCl_2_ or DMEM solution. Error bars represent SD. (**C**) Time-dependent ice crystal growth of the MgCl_2_ solution with 10% DMSO or nanopatch structures. Error bars represent SD. (**D**) Dynamic ice shaping (DIS) observation of Thr^96^-NP solution (200 ng/μl). A single ice crystal of approximately 10 μm in diameter made near the melting point about −0.03°C (top panel) and its growth is induced for 20 s (bottom panel). Scale bars, 10 μm. (**E**) Temperature profile of the microscope stage. The numbers indicate the time steps at which the snapshots were taken. (**F**) Wide-field fluorescence images of the nontreated cells and cells treated with NP and Thr^96^-NP structures (20 ng/μl) at five time steps. Green, sodium indicator. Scale bars, 100 μm. (**G**) Visually assessed cell viability of the control and nanopatch-treated cells based on the initial and final time steps (*n* = 155, 157, and 163 for the control, NP, and Thr^96^-NP groups, respectively).

Next, we investigated the mechanisms underlying the substantial ability of the peptide-functionalized DNA nanopatches to protect cells during cryopreservation. First, their extracellular IRI function was assessed using a splat cooling assay, which is a well-established procedure for quantifying the IRI effect by observing the growth of small ice crystals (Fig. 4A) (*16*, *41*). We deposited 20 μl of the sample liquid drop on the liquid nitrogen–cooled substrate, which resulted in the formation of tiny ice grains. After 30 min of incubation at −6°C, the average area of the 10 largest grains was calculated (Fig. 4B). DMSO solution (10%) diluted with 5 mM MgCl_2_ and DMEM buffer showed notable inhibition of ice crystal growth. The liquid-phase region observed in the 10% DMSO solution indicated that the freezing temperature of the solution decreased colligatively (Fig. 4A, second column panels). The IRI activity of the bare and peptide-functionalized DNA nanopatches was tested with either pristine (200 ng/μl in 5 mM MgCl*_2_*) or diluted conditions (20 ng/μl in DMEM). For comparison, the IRI activity of PVA, a well-known antifreezing polymer, was also tested with the same buffer conditions. All DNA nanopatches exhibited IRI activity comparable to PVA [weight-average molecular weight (*M*_w_): 9 kDa]; however, the extent of IRI in DMEM was less than 10% DMSO (Fig. 4B).

Time-dependent IRI measurements during the incubation at −6°C showed that bare and Thr^96^-NP structures exhibit somewhat lower IRI activity throughout the period (Fig. 4C). Considering the superior cryoprotective performance of the Thr^96^-NP over DMSO, this result implies that the IRI activity of a CPA does not necessarily reflect its contribution to cell cryopreservation. Several natural antifreezing proteins yield needle-like ice crystals owing to their binding characteristics with specific ice planes, which are considered to induce a negative effect on cell protection during cryopreservation (*42*). According to a dynamic ice shaping (DIS) test of the Thr^96^-NP solution (200 ng/μl), the ice crystal growth was isotropic, indicating that the DNA nanopatch did not exhibit such plane-specific binding characteristics (Fig. 4D). To verify the IRI activity of the DNA nanopatches, we performed all-atom molecular dynamics (AAMD) simulations (fig. S4A). Both the bare and Thr-functionalized DNA nanopatches inhibited the ice crystal growth during a 500-ns simulation (fig. S4, B and C), which was consistent with the experimental results. The number of hydrogen bonds (H-bonds) with the bare and Thr-NPs were similar, while their average lifetimes with ice molecules increased slightly in the Thr-NP design, especially at the Thr moieties (fig. S4, D and E).

Because intracellular ice formation and growth during cryopreservation produce detrimental effects on cell survival, ice growth within the cells was observed by fluorescence cryomicroscopy (*11*, *43*). Cells dyed using a sodium indicator were loaded on a cover glass and rapidly frozen by plunging the temperature of the cooling stage to −196°C and maintained for 2 hours. Intracellular ice grains are visible as black spots, as the dye molecules are expelled from the ice crystal. The evaluation of intracellular ice growth was performed during the temperature elevation to −6°C at a rate of 100°C/min (Fig. 4E). Subsequently, cells were incubated at −6°C for another 30 min and thawed. Notably, the “rapid-freeze and slow-thaw” profile used in this experiment is harsher than the typical “slow-freeze and rapid-thaw” cryopreservation process. Survival of the cells is more challenging, because small intracellular ice grains are apt to recrystallize during the incubation period at the subzero temperature. As a result, the Thr^96^-NP evidently induced the intracellular ice recrystallization to be retarded most over the control and bare NP cases upon thawing (Fig. 4F). After the cells were completely thawed at ambient temperature, the ratio of intact cells was classified by the shape and intensity of the remaining fluorescent signals. Cells not treated with any cryoprotectant showed a severe loss of fluorescent signals after the freeze-thaw cycle, indicating a substantial drop in the estimated intact cell ratio to as low as 9.0% (Fig. 4G). In contrast, cells incubated with the bare and Thr^96^-NPs (20 ng/μl) showed considerably better recovery rates (50.3 and 65.0%, respectively). The estimated intact cell ratio was in qualitative accordance with the cell viability results from the MTT assay.

### Deep learning–based cell survival analysis

**Fig. 5.**
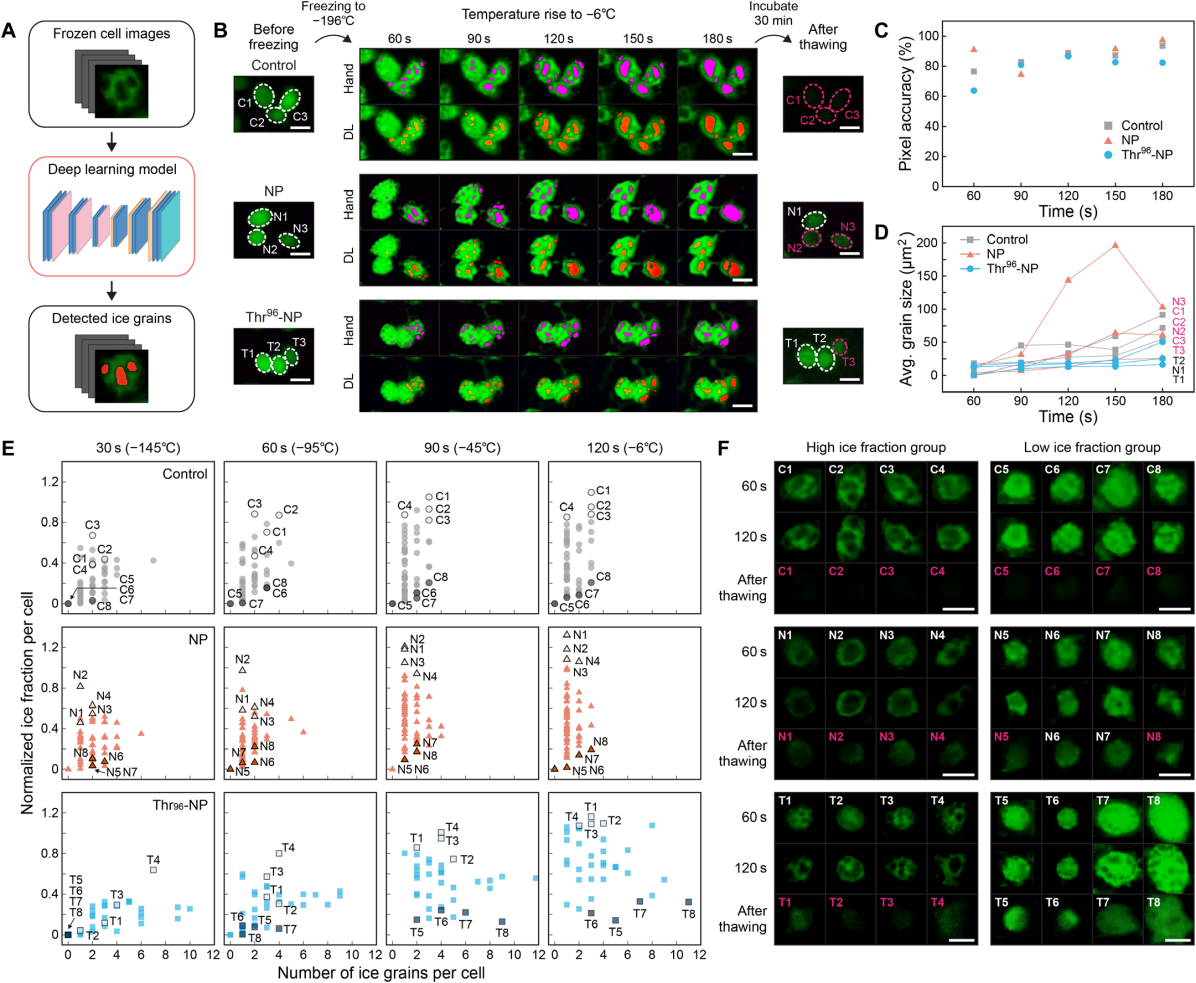
Deep learning–based intracellular ice grain analysis during the freeze-thaw cycle. (**A**) Schematic illustration of the deep learning–based ice grain detection process. (**B**) Fluorescence images of three representative cells for each group, showing the intracellular ice growth during the freeze-thaw cycle. Magenta-colored regions are the intracellular or near-cellular ice grains detected manually, and red-colored ones are those by the deep learning algorithm. DL, deep learning. Cells with a red dashed circle indicate that the fluorescent intensity was considerably reduced and the cell was not intact after thawing. Scale bars, 20 μm. (**C**) Pixel accuracy of ice grains detected by the deep learning model. Manually detected images were used as ground truth. (**D**) Overall area of intracellular ice grains per cell from the deep learning analysis. Cells denoted by pink-colored characters were shown to be damaged after thawing, as indicated in (B). (**E**) Distribution of the number and fraction of intracellular ice grains during the thawing process. Each data point corresponds to an individual cell. For each case, four representative cells with high and low ice fractions were selected to illustrate the evolution of intracellular ice grains during the thawing process. Refer to fig. S6A for the histograms of intracellular ice grain size (*n* = 56, 60, and 37 for the control, NP, and Thr^96^-NP cases, respectively). (**F**) Images of individual cells marked in (E) at 60 and 120 s and after thawing. Cells with high ice fraction present large intracellular ice grains and tend to show a substantial drop in fluorescent intensity after thawing, regardless of the treatment. Those with low ice fraction suppress the ice grain growth well, and Thr_96_-NP–treated cells showed a better conserved intensity after thawing. Cells denoted by pink-colored characters were shown to be damaged after thawing. Scale bars, 20 μm.

The fluorescent images revealed that the formation of large ice grains inside the cells appeared to be fatal for cell survival. However, the arbitrariness of defining the ice grain regions and laboriousness of tracking multiple cells at multiple time steps are critical limitations for further analysis of the dynamics and complex growth behavior of intracellular ice grains. We devised a breakthrough strategy to overcome such problems by developing a deep learning–based image recognition method to extract intracellular ice grains from fluorescent images (Fig. 5A). On the basis of the semantic segmentation method, the model was constructed using 605 individual cell images as training data to classify intracellular ice grains. From the result of three representative cells per case, we verified that our model successfully tracked intracellular ice grains compared to the result from intensity-based manual detection (Fig. 5B). The mean pixel accuracy of ice grains determined by the deep learning model and manual selection was 78.9% among different time steps (Fig. 5C). The fluorescence intensity of the cells with large intracellular ice grains tended to diminish after thawing, indicating that they were severely damaged, as denoted by pink-colored characters in Fig. 5 (B and D).

On the basis of this observation, we extended the deep learning–based analysis to detectable individual cells measured in Fig. 4F (fig. S5). After extracting the individual ice grains at different time steps, the probability histogram of the size of intracellular ice grains was assessed with time (fig. S6A). Subsequently, the area fraction of total ice grains normalized for each cell was tracked and correlated against the number of ice grains (Fig. 5E and fig. S6B). Here, the normalized ice fraction was calculated using the initial cell area measured at 30 s. During the thawing process, the ice fraction per cell generally tended to increase as the intracellular ice grains recrystallized, and thus the number decreased, which is simply observed for the control cells and those treated with the bare NP. However, the Thr^96^-NP, which yielded the highest cell viability after cryopreservation (Fig. 3C), showed a stark difference to the cell-thawing process such that the merge of the intracellular ice grains was noticeably retarded or suppressed (Fig. 5E, bottom row). To further investigate the cell survival in terms of the intracellular ice growth, we scrutinized selected cells that either exhibited high intracellular ice fractions (C1 to C4, N1 to N4, and T1 to T4 cells) or low ice fractions (C5 to C8, N5 to N8, and T5 to T8 cells) (Fig. 5, E and F). When completely thawed, every cell in the high ice fraction group led to poor recovery of fluorescence intensity (Fig. 5F, left panels). By contrast, the cells in the low ice fraction group ended up with a different fate; those treated with the Thr^96^-NP (T5 to T8) recovered well, but all control cells (C5 to C8) and the half of the NP-treated cells (N5 to N8) resulted in diminished recovery. Therefore, we suggest that the peptide-functionalized DNA nanopatches not only suppress the intracellular ice growth but also contribute further by providing another protective function for improving the cell viability.

### Protection of cell membrane

**Fig. 6.**
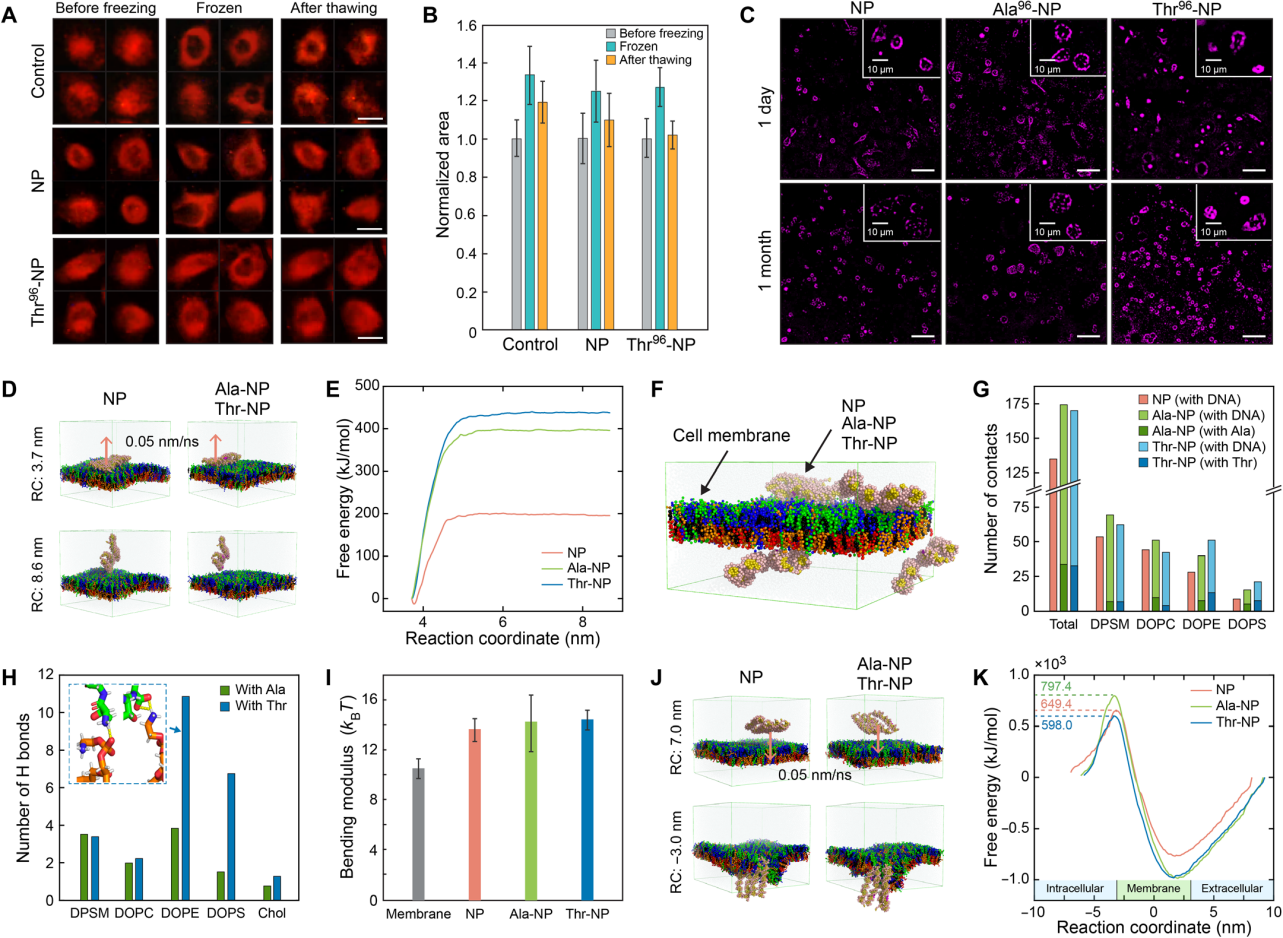
Analyses of the cell membrane protection effect of DNA nanopatches. (**A**) Fluorescent images of representative cells showing the deformation of the cells during the freeze-thaw process. Scale bars, 20 μm. (**B**) Change and recovery in geometry of nanopatch-treated cells during the freeze-thaw process (*n* = 152, 123, and 126 for the control, NP, and Thr^96^-NP cases, respectively). Error bars represent SD. (**C**) Fluorescent images of thawed cells after cryopreservation in liquid nitrogen temperature for 1 day and 1 month, showing the existence of DNA nanopatch structures. Magenta, Cy3 moiety on NP structures. Scale bars, 50 μm. (**D**) Snapshots of umbrella sampling simulations for calculating binding energy of miniaturized NP structures with lipid membranes. RC, reaction coordinate. (**E**) Free energy profiles for NP, Ala-NP, and Thr-NP binding to the bilayer surface as a function of the reaction coordinate, the *z* axial distance between NPs, and bilayer centers. (**F**) Final configurations of lipid membranes with four NP structures. The Egg sphingomyelin (DPSM), DOPC, DOPE, and 1,2-dioleoyl-sn-glycero-3-phospho-L-serine (DOPS) lipids and cholesterols are represented by the green, blue, orange, red, and black spheres, respectively. (**G**) Comparison of average numbers of contact between moieties of NPs and lipid components among membranes calculated during the last 500 ns of the CGMD simulation. (**H**) Number of H bonds between lipid and peptide molecules calculated during the last 10 ns of the AAMD simulation. Enlarged view of H-bonds formed between the amine or phosphate group of DOPE and Thr was shown in the inset. (**I**) The averaged values of bending modulus over the last 0.1 μs of the CGMD simulations. Error bars represent SD. (**J**) Snapshots of umbrella sampling simulations of the endocytosis pathway. (**K**) Free energy profiles for endocytosis pathways of NPs as a function of the reaction coordinate.

Another threat to cell survival during cryopreservation is cell deformation due to ice crystal growth. In this regard, the encapsulation of cells with nanomaterials has been attempted for protection against extracellular ice crystals (*44*, *45*). When exposed to the similar freeze-thaw profile as in the intracellular ice grain measurement, the distortion of frozen cells was frequently observed (Fig. 6A). The extent of cell distortion and recovery compared with the initial state was further assessed (Fig. 6B). The average area of cells that experienced freezing and recrystallization increased by 33% in DMEM (control) and ~26% when the nanopatches were treated. After thawing completely, the increase in area was reduced to 19 and 9% for the control and NP, respectively; however, Thr_96_-NP noticeably limited the increase in area at 2%, indicating that initial cell geometry is nearly recovered. We found that the DNA nanopatches remained around and inside the cells even after 1 month of cryopreservation (Fig. 6C). The fluorescent intensity of the Thr_96_-NP was higher than those of the bare NP and Ala_96_-NP, which is consistent with the fluorescent images before freezing in Fig. 3 (A and B). From the observation that (i) the DNA-peptide nanopatches can be attached to the cell membrane throughout the long-term cryopreservation and (ii) the conservation of the membrane against ice crystal growth through freeze-thaw process is another important phenomenon from the surviving cells, we explored the possibility that the nanopatches interacting with the cell could enhance the ability to withstand its severe membrane distortion.

To elucidate the protective mechanism acting on the membrane, coarse-grained MD (CGMD) simulations of DNA nanopatches on the realistic membrane were performed. We conducted a series of steered MD (SMD) simulations to calculate the binding free energy between the DNA nanopatch and the cell membrane (*46*). The miniaturized DNA nanopatch used in the simulation consisted of four 32–base pair–long dsDNA helices. For Ala- and Thr-functionalized DNA nanopatches, four peptide chains (five consecutive Ala or Thr) were attached to the bare NP. The DNA nanopatches were initially bound to the bilayer and were pulled from the bilayer at a constant velocity of 0.05 nm/ns (Fig. 6D). The binding free energy of the nanopatches was in the order of Thr-NP (438 kJ/mol), Ala-NP (397 kJ/mol), and bare NP (195 kJ/mol) (Fig. 6E). The DNA-peptide nanopatches, especially Thr-NP, showed more than twofold increase over the bare NP, indicating that the former had a stronger interaction with the cell membrane. This result is consistent with the experiments, showing that the Thr^96^-NP was better attached to the cells than others.

We further performed CGMD simulation with four miniaturized nanopatches of each design, placed close to both leaflets of the membranes (Fig. 6F), to investigate the molecular interactions involved. The DNA nanopatches bound to the cell membrane during the simulation, and the interactions between the moieties of the nanopatches and the lipid molecules of the membrane were monitored by measuring the average number of contacts between them over the last 500 ns of the simulation (Fig. 6G). The Ala and Thr moieties, which preferentially interacted with 1,2-dioleoyl-sn-glycero-3-phosphocholine (DOPC) and 1,2-dioleoyl-sn-glycero-3-phosphoethanolamine (DOPE) lipid molecules, increased the number of total intercontacts, causing a stronger binding energy with the cell membrane. We conducted another AAMD simulation with free Ala and Thr molecules on the cell membrane to compare their interactions with lipid molecules in more detail (Fig. 6H). The analysis of H-bonds showed that Thr formed more H-bonds than Ala, especially with DOPE, since DOPE has both H-bond donors and acceptors interacting with hydroxyl and amine groups of Thr.

To understand the effects of the nanopatch attachment on the membrane stiffness, the bending modulus of the membrane was calculated from the power spectra of height fluctuation of phosphate beads at the lipid bilayer (*47*). The bending modulus was averaged over the last 0.1 μs, yielding 10.6 ± 1.6 *k*_B_*T* for the membrane without any DNA nanopatch (Fig. 6I). The average bending modulus was higher for the membrane with bare NP (13.7 ± 1.7 *k*_B_*T*), Ala-NP (14.2 ± 4.7 *k*_B_*T*), and Thr-NP (14.8 ± 1.8 *k*_B_*T*), showing a stiffer bilayer in the presence of the DNA nanopatches. Together, the Thr residues expressed on the DNA nanopatch surfaces play a critical role in increasing membrane stiffness by interacting strongly with the membrane, thereby contributing to the increase in cell viability during cryopreservation. A series of SMD simulations were further performed to compare the endocytosis pathways of the bare, Ala-, and Thr-NPs. The respective nanopatch was initially located outside the bilayer and was pushed into the bilayer at a constant velocity of 0.05 nm/ns (Fig. 6J). From the free energy landscape, an energy barrier of entry through the bilayer for the Thr-NP (598.0 kJ/mol) was lower than those of the bare NP (649.4 kJ/mol) and Ala-NP (797.4 kJ/mol), respectively, by 7.9 and 25.0% (Fig. 6K). Considering the fact that the Thr-NP structures were populated higher near the cell membrane, it is plausible that the extent of their endocytosis subsequently intensified.

## DISCUSSION

In summary, we found that the Thr-functionalized DNA nanopatch resulted in a remarkable cryoprotection of HSC-3 cells, compared with 10% DMSO, in terms of (i) a considerably lower working concentration range, (ii) higher cell viability tested up to 1 month of storage, and (iii) minimal dependence on the freezing temperature profiles. Such distinctive cryoprotection characteristics of the Thr-DNA nanopatches over other CPAs originate from the synergetic activity of ice growth retardation and cell membrane protection. When incubated for approximately 2 hours, these nanopatches were sufficiently attached to the cell membrane and thus well internalized into the cytoplasm of target cells, enabling efficient cell cryopreservation upon the introduction of DNA nanopatches (30 ng/μl, ~5.3 nM) in the cell buffer. Functionalization of the bare DNA nanopatch with the antifreezing peptides, especially Thr, enhanced the interactivity of the patches to the cells, as validated by confocal microscopy and CGMD simulation. This unique effect of the Thr moiety, in addition to its inherent IRI ability, contributes to its superior long-term cryopreservation performance. Our experiments, including MD simulations, revealed that the DNA-peptide nanopatches exert multisite actions that result in a synergetic effect, which protects the cells throughout the freeze-thaw process.

The substantial growth of intracellular ice, enlarged from the intracellular seeds or propagated from the extracellular region, is known to be lethal. Therefore, it should be mitigated during cryopreservation to improve cell survival (*5*, *48*). The DNA nanopatches efficiently retarded intracellular ice growth and recovered membrane geometry, resulting in successful cryopreservation of cells after the freeze-thaw cycle. Colligative membrane-permeable CPAs such as DMSO can suppress intracellular ice formation and growth; however, they typically require optimized cooling rates for controlling the outflux of water molecules inside the cells. The Thr-DNA nanopatches exhibited outstanding cryoprotection ability, regardless of the freezing conditions.

DNA nanostructures within the cells or cell culture medium can be eventually disassembled and digested, mainly due to the depletion of Mg^2+^ ions and nuclease activity (*49*). In addition, they have not exhibited cytotoxicity within the typical concentration range used in this study (*30*, *31*, *49*, *50*). The biocompatibility and biodegradability of DNA-based nanomaterials could be advantageous for their use as cell cryoprotectants for replacing other cytotoxic CPAs by reducing the side effect of remaining CPAs and obviating the need of washing process that can induce the loss of thawed cells. Despite the remarkable cell cryopreservation ability of the Thr-DNA nanopatch reported in this study, there is room for further enhancement of its performance by optimizing its geometry and mechanical properties such as cross-sectional design, surface-to-volume ratio, and structural rigidity. Considering that the endocytosis efficiency of DNA nanostructures is influenced by their geometries and cell types (*30*), the shape and function of cryoprotective DNA-peptide assemblies can be tailored for targeting specific cells by using the design versatility and programmability of structural DNA nanotechnology. The type, density, and arrangement of antifreezing peptides on DNA nanostructure are important design factors for its cryoprotective function as well. In addition, our system can have the potential to integrate other biological functions of DNA nanostructures such as intracellular delivery and receptor-specific binding mechanisms (*28*, *51*). In terms of cost-effective and mass production of cryoprotective DNA-based nanostructures, bacteriophage-based production of DNA can be adopted to markedly reduce the fabrication cost (*52*). It can lead to the practical use of DNA-based cryoprotective materials while providing excellent cryoprotective performance. With advanced designs and production methods based on the diverse understanding of cryoprotection mechanisms, we expect that the use of cryoprotective DNA nanostructures can be extended to the preservation of higher-order biological systems such as embryos, cell spheroids, and tissues.

## MATERIALS AND METHODS

### Preparation of DNA nanostructures

A folding mixture (100 μl) was prepared that contained scaffold DNA (30 nM), staple strand (100 nM each), 1× TAE buffer (40 mM tris-acetate and 1 mM EDTA), and MgCl_2_ (12 to 20 mM). The sequence of all staple strands is summarized in tables S1 to S4. For the self-assembly of Ala- and Thr-DNA nanopatches, 5× excess PNA strands regarding the scaffold concentration and the number of handles were added to the solution. The final mixture was subjected to the following temperature gradient using a thermocycler (T100, Bio-Rad, USA): heated to 80°C at 1°C per second, cooled down from 80° to 65°C in 1 hour (2 min per −0.5°C) and 65° to 25°C in 40 hours (30 min per −0.5°C), and maintained at 4°C. After self-assembly, excessive nucleic acid strands were removed by centrifugal filtration using Amicon Ultra Centrifugal Filter Units with 50-kDa cutoff filters (UFC505096, Merck KgaA, USA). The concentrations of the purified DNA origami nanostructures were set to 100, 200, and 300 ng/μl using a microvolume ultraviolet-visible spectrophotometer (NanoDrop One, Thermo Fisher Scientific, USA). We assumed that the 260-nm absorbance of the DNA-PNA duplex was identical to that of the DNA duplex.

### Splat cooling assay

The IRI activity of samples was assessed via the splat cooling method. A polarized optical microscope (U-MSSPG, Olympus, Japan) and a nanoliter osmometer (Otago Osmometers Ltd., New Zealand) cooling stage were used. To determine the IRI activity, 20 μl of the solution containing DNA nanostructures was dropped from a height of 1.5 m onto the surface of a cleaned glass placed on the liquid nitrogen–cooled metal island. As the temperature of the metal island was set to −150°C, the droplet froze instantly and formed a thin solid ice film. Subsequently, the temperature of the glass was increased to −6°C by moving onto the precooled nanoliter osmometer cold stage. Subsequently, the ice film was annealed at this temperature for 30 min for evaluating the IRI activity. Next, the ice wafer was imaged using a digital camera (U-MSSPG, Olympus) fitted to the microscope for determination of the grain sizes of the ice crystals. Image processing was conducted using the ImageJ software (*53*). Ten of the largest ice grain domains in the field of view were chosen and averaged to evaluate IRI activity. The average results from three individual experiments for each sample were used.

### DIS assay

To investigate the effects of the DNA nanopatches on ice growth, the growth of a single ice crystal in the aqueous dispersion was studied using a nanoliter osmometer (Otago Osmometers Ltd.). Before the samples were measured, the device was calibrated using ultrapure water and NaCl solution (0.05 mol/kg) to obtain the “zero” and “−0.18°C” points, respectively. To observe the growth of the single ice crystal, we injected a submicroliter volume of DNA nanostructure dispersion into a temperature-controlled sample holder that was 500 μm in diameter and 1 mm in depth. The sample holder was quickly frozen and then slowly warmed to its melting temperature. Once a small (approximately 10 μm in diameter) single ice crystal appeared, it was maintained for approximately 20 s at a constant temperature.

### MTT assay for HSC-3 cells

Mitochondrial activity was assessed by MTT assay using spectrophotometry. The principle of this method is based on the reduction of MTT to formazan crystals by the mitochondrial succinyl dehydrogenase enzyme, which is active only in living cells. Immediately after warming, HSC-3 cells were homogenized in PBS and incubated with 10 μl of MTT solution (100 μg/ml) that was added to the HSC-3 culture (90 μl, 5 × 10^5^ cells). After incubation at 37°C for 2 hours, the medium was discarded. For each sample, 100 μl of 2% SDS solution (100 μl of 10% in 0.01 M HCl) was added for solubilization and incubated at 37°C for 4 hours, without the addition of DMSO. Absorbance at 570 nm was measured using a microplate reader. Fresh DMEM (200 μl) without HSC-3 cells was used as a control. HSC-3 cells cryopreserved in 10% DMSO freezing media were considered to be 100% viable.

### AAMD simulation

The molecular structure of the DNA nanopatch was generated by Structured NUcleic acids Programming Interface (SNUPI) (*54*). The AAMD simulations were performed via GROMACS 5.1.4 (*55*) using the CHARMM36 force field (*56*). A leapfrog integrator was used to conduct all AAMD simulations with a time step of 2 fs. A V-rescale thermostat (*57*) was used to control the temperature of the simulations at 267.15 K. The pressure was maintained at 1 bar during the simulations using the Berendsen (*58*) and Parrinello-Rahman (*59*) barostats for the equilibrium and production runs, respectively. A Verlet cutoff with a range of 1.2 nm was used to build neighbor lists, which were updated at every step of the simulations. Particle Mesh Ewald (PME) with a cutoff of 1.2 nm was used to calculate electrostatic interactions (*60*). The linear constraint solver (LINCS) algorithm was used to constrain the bond lengths during the simulations (*61*). The structure and force field of the DNA nanopatch were built using the CHARMM-GUI PDB reader (*62*). Thr modifications were made on the basis of the experimental design positions. Parameters of the Thr were obtained from CHARMM36, and parameters connecting Thr and DNA were derived with CGenFF (*63*).

To elucidate the IRI effects of bare and Thr-NPs, continuous ice growth systems with the TIP4P/ICE water model (*64*) were built in a simulation box of 16.2, 14.2, and 11.2 nm. Seed ice was inserted at the bottom of the system for ice growth. Disordered water molecules with fixed positions were inserted below the seed ice to prevent the downward growth of ice. All systems were ionized with 20 mM Mg^2+^ and Cl^−^ ions to represent the experimental conditions. Overall charge neutrality was achieved by adding Mg^2+^ ions. For the AAMD simulation of Thr moieties on the cell membrane, the initial parameters and the system were constructed on the basis of the previous report (*65*). GROMACS utilities were used for analyses, including hydrogen bonding lifetime and *z*-axis directional movement analyses. PyMOL (PyMOL Molecular Graphics System version 2.0 Schrödinger LLC) was used for visualizing the AAMD simulation results. For the hydrogen-bonding lifetime analyses, the lifetime between DNA patches and water/ice molecules was integrated in the period of 490- and 500-ns simulation time by accumulating all the H-bonds formed between the amine, carbonyl, and hydroxyl groups. *Z*-axis directional movement analyses were performed for the entire 500 ns of the simulations.

### CGMD simulation

All atomic structures of bare, Ala-DNA, and Thr-DNA nanopatches were converted to the CG model by the modified version of “martinize.py” with the Martini DNA force field (*66*). CG simulations were conducted using a leapfrog integrator with a time step of 10 fs. The temperature and pressure of the CG systems were maintained at 310.15 K and 1 bar using a V-rescale thermostat and Berendsen barostat, respectively. A Verlet cutoff scheme with a buffer tolerance of 0.005 kJ/mol was used to update the neighbor lists every 20 steps. The reaction field method with a cutoff range of 1.1 nm and a dielectric constant of 15 was used to evaluate the Coulomb interactions. For van der Waals interactions, a cutoff range of 1.1 nm was used. The LINCS algorithm was adopted to constrain the bond lengths. To illustrate the effects of DNA nanopatches on cellular membranes, a model of a realistic plasma membrane (*67*) was used with four bare or Thr-DNA nanopatches placed close to both leaflets. All membrane systems were solvated with at least 25 water beads per lipid and ionized with 150 mM Na^+^ and Cl^−^ ions. Overall charge neutrality was achieved by adding Na^+^ ions. The final dimensions of the simulation box were 24.7, 24.7, and 15.1 nm.

### Analysis details for CGMD simulation

The Python code generated by Fowler *et al*. (*47*) was used to calculate the bending modulus of the membrane. The power spectra of height fluctuations were calculated using the equation *q*^4^⟨∣*h*(*q*)∣^2^⟩ = *k*_B_*T*/*K*_c_, based on the Helfrich-Canham theory (*68*) for the interval of 0.1 μs. Because this expression is only valid at small wave numbers, the least squares method was applied for points in *q* < 0.6 nm^−1^ to obtain the bending modulus, *K*_c_.

To calculate the binding energy between bare, Ala-DNA, or Thr-DNA nanopatches and the membrane, we performed umbrella sampling to pull the nanopatch from the membrane. Initially, a single DNA nanopatch was inserted close to the membrane systems and equilibrated for 100 ns to construct the initial configurations for umbrella sampling. For generation of the *z*-directional reaction coordinate, it was pulled from the membrane surface by the pulling code with a force constant of 1000 kJ mol^−1^ nm^−2^. For both systems, 50 windows with a spacing of 0.1 nm to cover the range from 3.7 to 8.6 nm were created for calculating the binding energy. Each window was equilibrated for 1 ns and subsequently subjected to a 5-ns production run. The weighted histogram analysis method (WHAM) tools in the GROMACS package were used to calculate the energy profile of the pulling simulations.

The free energy profile of the endocytosis pathway of bare, Ala-DNA, or Thr-DNA nanopatches was also determined on the basis of umbrella sampling. A DNA nanopatch was placed above the membrane, with the minimum distance between the membrane and it set to 6 to 7 nm. A pulling code with a force constant of 500 kJ mol^−1^ nm^−2^ was used for generating the *z*-directional reaction coordinate with a distance of 15 nm. We equilibrated 150 windows with a spacing of 0.1 nm for 1 ns and performed a 10-ns production run for each window. The WHAM tools in the GROMACS package were also used for determining the free energy profiles of the endocytosis pathway.

### Deep learning–based intracellular ice grain analysis

We adopted a deep learning–based semantic segmentation model (*69*) to automatically extract ice grains from cropped individual cell images. The binary cross entropy loss (*L*_BCE_), defined as $L_{\text{BCE}}(y,\hat{y})=-\{y\text{log}(\hat{y})+(1-y)\text{log}(1-\hat{y})\}$, was used to calculate the degree of difference between the ice image from the model and the ground truth. Here, *y* and $\hat{y}$ represent the ground truth and the predicted ice image from the model, respectively. We also introduced the dice loss (*L*_D_), defined as $L_{D}(y,\hat{y})=1-(2y\hat{y}+1)/(y+\hat{y}+1)$, to consider the degree of overlap with the ground truth image and measure the similarity between the extracted and the ground truth ice images. Then, the total loss function was defined as *L* = 1/*N* ∑ (*L*_BCE_/2 + *L*_D_/2) with the batch size *N*. In total, 605 individual cell images captured at different time steps of the recorded videos were used to train the model. They were divided into the training and validation sets (591 and 14 images, respectively). Every image was augmented by eightfold via parallel flip, vertical flip, and rotation (0°, 90°, 180°, and 270°). The learning process was performed with *N* = 10 using the Adam optimizer (*70*) on a personal computer with Titan RTX 2070. It took 189.3 s to analyze 3232 individual cell images.

### Statistical analysis

The methods for statistical analysis and sample sizes (*n*) are specified in each figure legend, including the definitions of error bars. Statistical significance was assessed using an independent *t* test with 95% confidence intervals using Statistical Package for the Social Sciences Statistics 25.
