# Supplementary material for: Peptide-DNA origami as a cryoprotectant for cell preservation
Source: Sci Adv. 2022 Oct 28;8(43):eadd0185. doi: 10.1126/sciadv.add0185 (PMC9616499; doi:10.1126/sciadv.add0185)
Supplement: Supplementary file 1 — Supplementary Text Figs. S1 to S6 Tables S1 to S5 [file sciadv.add0185_sm.pdf]

Science Advances
AAAS

Supplementary Materials for

Peptide-DNA origami as a cryoprotectant for cell preservation

Chanseok Lee *et al.*

Corresponding author: Dong June Ahn, ahn@korea.ac.kr; Do-Nyun Kim, dnkim@snu.ac.kr

Sci. Adv. **8**, eadd0185 (2022)
DOI: 10.1126/sciadv.add0185

This PDF file includes:

Supplementary Text
Figs. S1 to S6
Tables S1 to S5

Supplementary Text

Agarose gel electrophoresis

Samples were electrophoresed on 1.5% agarose gels containing 0.5× TBE and 12 mM $MgCl_2$. The loaded samples were allowed to migrate for 1.5 h at 75 V bias voltage (approximately 3.7 V/cm) in an ice-water-cooled chamber (i-Myrun; Cosmo Bio Co. Ltd., Japan). The gels were stained using 0.5 μg/mL ethidium bromide (EtBr) solution (Noble Bioscience Inc., Korea), and imaging was performed using the GelDoc XR+ device and the Image Lab v5.1 program (Bio-Rad).

AFM measurement

Assembled DNA origami structures were diluted with the annealing buffer to approximately 0.5–1 nM prior to the measurement. The prepared sample (20 μL) was deposited on a freshly cleaved mica substrate (highest grade V1 AFM Mica; Ted-Pella Inc., USA) and subsequently incubated for 5 min. The substrate was washed with DI water three times and gently dried using a N_2 gun (<0.1 Kgf/cm^2). AFM images were obtained using NX10 (Park Systems, Korea) and a PPP-NCHR probe with a spring constant of 42 N/m (Nanosensors, Switzerland). The non-contact mode was used to measure typically 5 μm × 5 μm of the sample area at 1024 × 1024 pixel resolution using the SmartScan software. All measured images were flattened in linear and quadratic order using the XEI 4.1.0 program (Park Systems).

Cell culture

Human oral squamous cell carcinoma (HSC-3) cells were purchased from Sigma-Aldrich (USA). Cell passages between 8 and 10 were used for all experiments. Cells were grown in Dulbecco's modified Eagle's medium (DMEM)-glucose medium (Gibco, USA) supplemented with 10% fetal bovine serum (FBS) (heat inactivated, Gibco) and 100 U/mL penicillin–streptomycin (Gibco). All cell lines were plated in 75 cm^2 tissue culture flasks and cultured at 37°C in a humidified atmosphere under 5% CO_2. The cells were sub-cultured every 48–72 h by detaching them with trypsin–EDTA (Gibco) and resuspending them in a 75 cm^2 culture flask containing 15 mL of culture medium.

Treatment of HSC-3 cells with DNA nanopatches

HSC-3 cells were respectively subcultured in 14 mm diameter plates and allowed to grow for 24–48 h. Before incubation with the DNA nanopatches, cells (5.2×10^5 cells/mL) were washed twice with fresh media. Cells were incubated with the DNA nanopatch solution for 0 min, 30 min, 1 h, 2 h, 4 h, and 8 h at 37°C under humidified 5% CO_2.

HSC-3 cell staining

We prepared a stock solution of Hoechst 33258 (maximum emission at 461 nm) by dissolving the solid in DI water to obtain concentrated stock solutions of 1 mg/mL, which were stored at 4°C, protected from light, and warmed to room temperature before use. Since the HSC-3 cell is an adherent cell, it can be stained in situ on coverslips. Hoechst 33258 stock solution was added to 1 mL of fresh media (final concentration of 1 μg/mL) and incubated for 10 min at 37°C in a humidified 5% CO_2 atmosphere. A stock solution of sodium indicator (CoroNa™ Green, maximum emission at 516 nm) was prepared by dissolving the solid in DMSO, resulting in a concentrated 1 mg/mL stock solution, which was stored at −20°C, protected from light, and warmed to room temperature before use. The sodium indicator stock solution was added to 1 mL of fresh media (final concentration of 1 μM) and incubated for 15 min at 37°C in a humidified atmosphere under 5% CO_2. A stock solution of TagRFP (CellBrite™ Red, maximum emission at 665 nm) was stored at 4°C, protected from light, and warmed to room temperature before use. TagRFP solution (5 μL) was added to 1 mL of fresh media and incubated for 60 min at 37°C in a humidified atmosphere under 5% CO_2. After incubation, the staining medium was removed, and the cells were washed with fresh warm growth medium and incubated at 37°C for 60 min.

Confocal microscopy

Confocal microscopy was performed using an LSM700 microscope (Carl Zeiss, Germany) with 405, 488, and 555 nm lasers. Images of the cells incubated with various DNA nanopatches on coverslips were obtained at 10× magnification and 2048 × 2048 pixel resolution. For the z-stack measurements, 20 images were taken sequentially. Calibration and channel merging of the raw images were performed using the ImageJ software.

Cell cryopreservation

Live HSC-3 cells cultured in a 75T cell flask were detached from the bottom by the addition of EDTA. The cells in suspension were transferred to a 15 mL conical tube and pelleted by centrifugation for exchanging the solvent with fresh media. DMSO or DNA nanopatch solution (5 μL) was mixed with 45 μL of a solution containing 1.5×10^6 cells with fresh media. Cells with DNA nanopatches were incubated for a designated period at 37°C under humidified 5% CO_2 prior to freezing. In the case of slow freezing, the cells were frozen for 12 h at −40°C in a cell-freezing container (Mr. Frosty filled with isopropyl alcohol). They were subsequently transferred to a liquid nitrogen chamber and subjected to cryopreservation (1 day, 1 week, and 1 month).

Fluorescence cell imaging after thawing

We first removed the HSC-3 cells stored in a liquid nitrogen chamber and quickly thawed them at 37°C. Pre-heated fresh media (37°C, 1 mL) was added to each sample and the pellet was resuspended by pipetting. Thawed HSC-3 cells were dispensed into a Petri dish containing 3 mL of pre-heated fresh media and a coverslip. After 3 h of incubation at 37°C under humidified 5% CO_2, the coverslip was washed with fresh media to remove damaged and non-attached cells. Subsequently, attached cells were incubated for 24 h before observation. Cell imaging was performed in PBS buffer after live-cell staining using a sodium indicator to avoid interference by autofluorescence.

Fluorescence cryomicroscopy for intracellular ice-crystal observation

To observe the growth of intracellular ice crystals, HSC-3 cells were sandwiched between two cover glasses in the presence of fresh media and placed on a cold stage (THMS600; Linkam, UK). The samples were cooled at a rate of 100°C/min, maintained at −196°C for 2 h, and then heated to −6°C at a rate of 100°C/min to observe induced intracellular ice recrystallization. Subsequently, the sample was annealed at −6°C for 30 min and thawed.

Fluorescence imaging of DNA nanopatches after cryopreservation

HSC-3 cells stored in a one day or one month in the liquid nitrogen chamber were quickly thawed at 37°C. Pre-heated fresh media (37°C, 1 mL) was added to each sample and the pellet was resuspended by pipetting. Thawed HSC-3 cells were dispensed into a Petri dish containing 3 mL of pre-heated fresh media and a coverslip. After 3 h of incubation at 37°C under humidified 5% CO_2, imaging was performed in PBS buffer without cell staining to observe Cy3 dye on the DNA nanopatches.

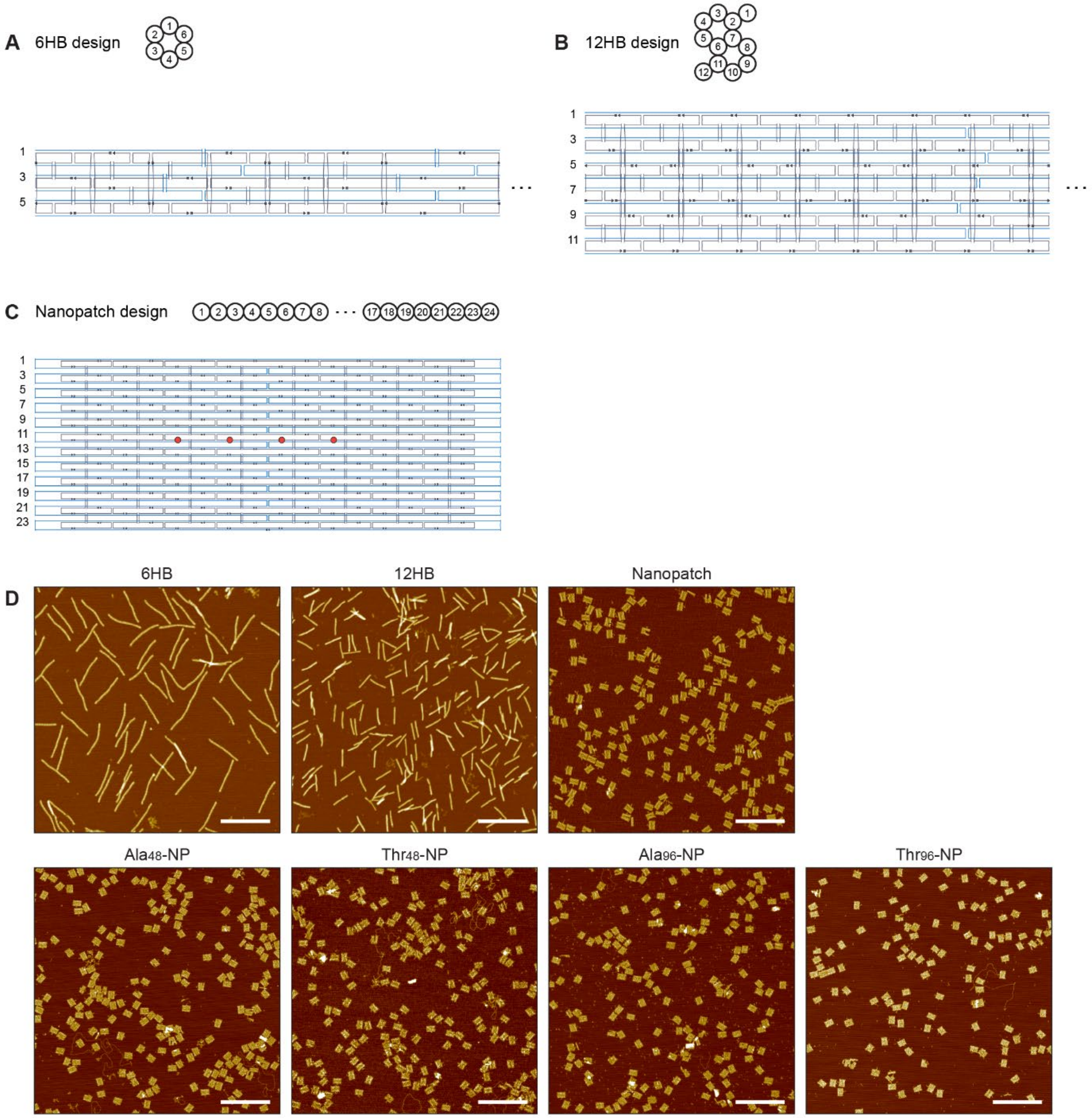

Fig. S1. Design and fabrication of cryoprotective DNA nanostructures. (A and B) Cross-sectional design and Cadnano diagrams showing the repetitive scaffold and staple route of the (A) 6HB and (B) 12HB design. Blue lines indicate the scaffold and gray lines indicate the staple strands. Refer to Table S1 and S2 for DNA sequences. (C) Cross-sectional design and cadnano diagrams showing the scaffold and staple route of the nanopatch design. Red circles indicate the Cy3 dye positions. Refer to Table S3 and S4 for DNA sequences. (D) Large-area AFM images of various cryoprotective DNA origami nanostructures. Scale bars: 500 nm.

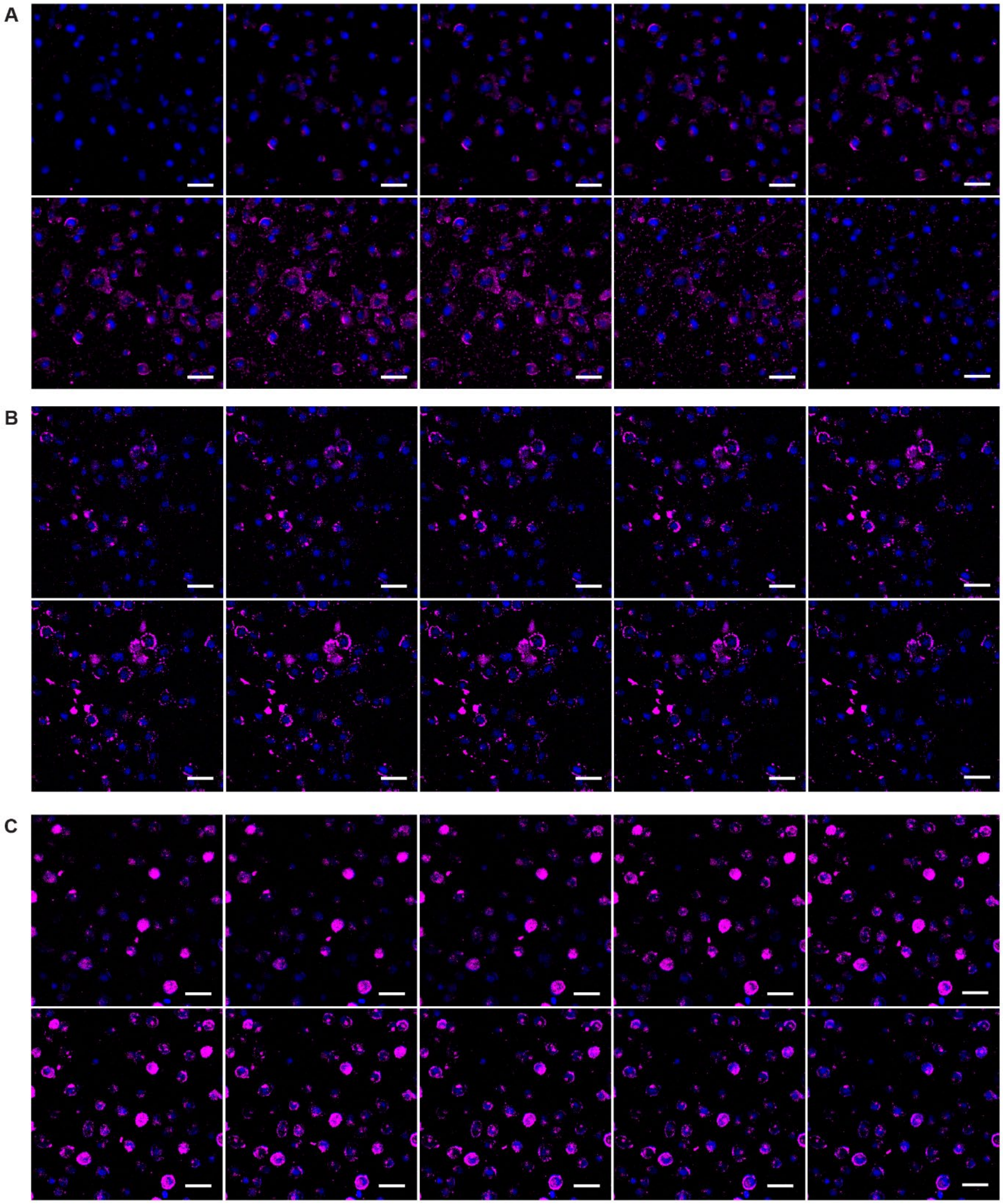

Fig. S2. Z-stack confocal microscope images of the HSC-3 cells treated with bare and peptide-functionalized nanopatch structures. Cells were treated with 20 ng/uL of the (A) bare NP, (B) Ala_{96}-NP, and (C) Thr_{96}-NP for 2 h. Scale bars: 50 μm.

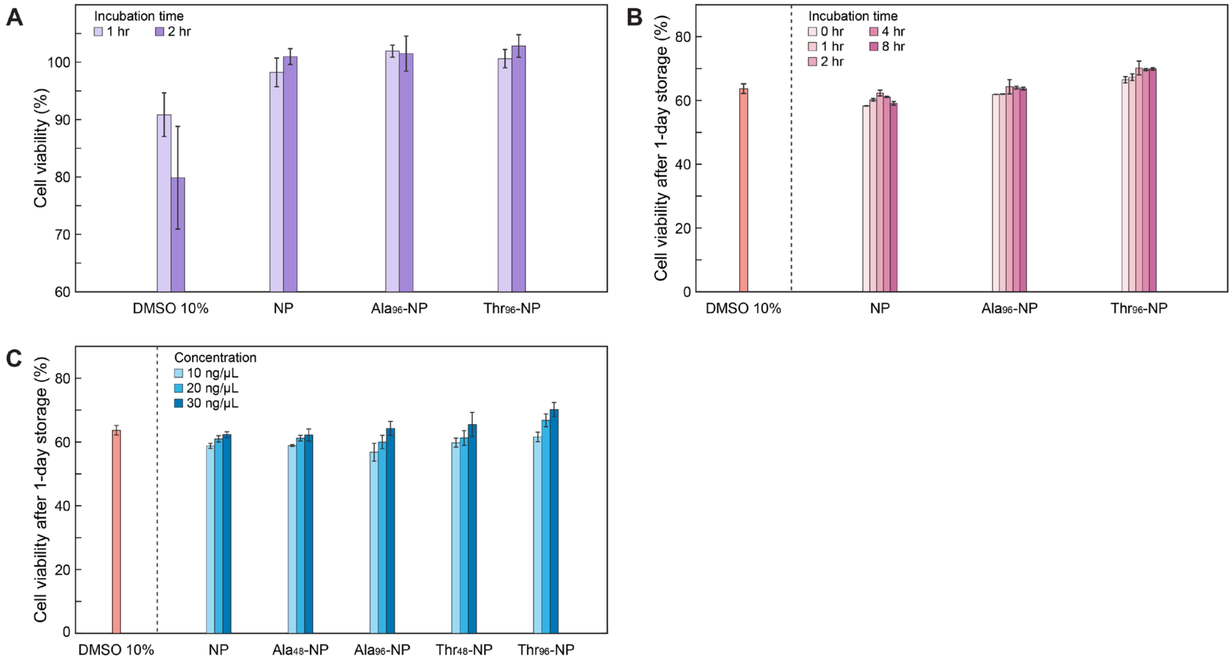

Fig. S3. Additional MTT assay results with respect to incubation time and nanopatch concentration. (A) MTT assay for the cells incubated with 10% DMSO and 30 ng/μL of bare and peptide-functionalized nanopatches for 1 and 2 h. Cell viability was measured without cryopreservation process. (B) MTT assay for the cells treated with 30 ng/μL of bare and peptide-functionalized nanopatch structures with different incubation time up to 8 h, and after stored in a liquid nitrogen chamber for one day. (C) MTT assay for the cells treated with various concentrations of bare and peptide-functionalized nanopatch structures for 2 h, and after stored in a liquid nitrogen for one day.

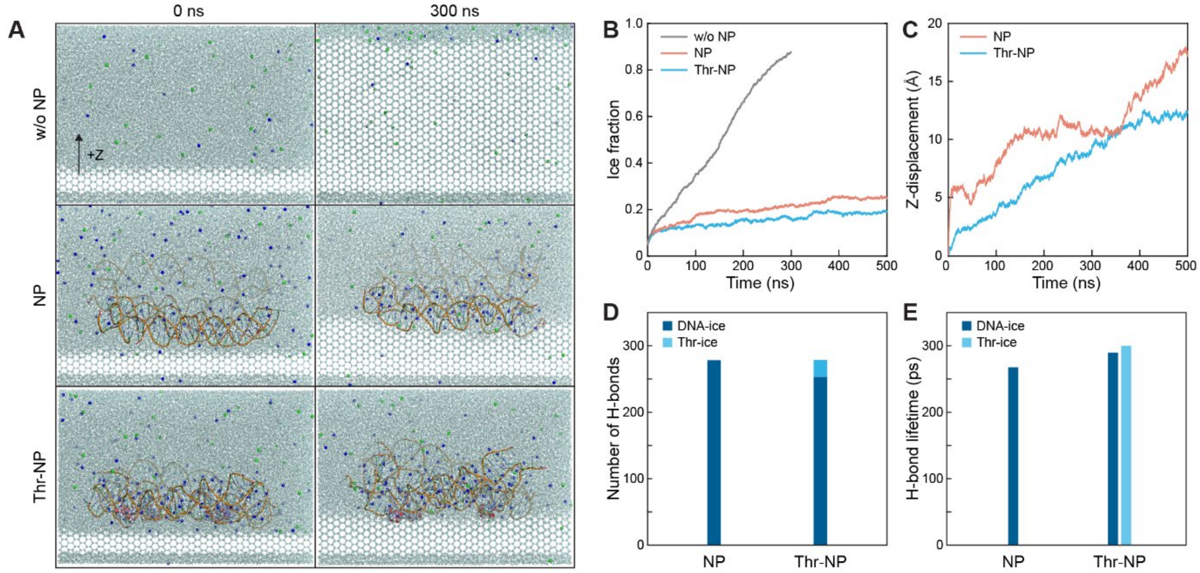

Fig. S4. AAMD simulation to investigate the underlying IRI mechanism of DNA nanopatches. Water boxes containing 20 mM $MgCl_2$ were constructed, and the initial seed ice crystals were inserted at the bottom of the boxes. A miniaturized DNA nanopatch structure consisting of four 84 bp dsDNA helices was constructed, and four 5-mer threonine residues were attached to the Thr-DNA nanopatch design. (A) MD simulation snapshots indicating the ice crystal growth at the initial stage and after 300 ns of simulation time. Miniaturized NP and Thr-NP structures showed noticeable IRI activity. (B) The ratio of water molecules at the ice phase during the freezing simulation. The fraction of ice in the system without nanopatches increased immediately and reached 0.87 after 300 ns of simulation; in systems with the bare and Thr-DNA nanopatch structures, the fraction of ice increased more slowly and reached 0.25 and 0.19 after a 500 ns simulation, respectively. (C) Z-directional movement of DNA nanopatch structures during the freezing simulation. The threonine modification contributed to the IRI activity to a limited degree. (D) The average number of hydrogen bonds (H-bonds) between DNA patches and secondary prism plane of ice, which are calculated by dividing the number of hydrogen bonds between DNA or threonine moieties and ice. (E) Calculated H-bond lifetime of DNA and threonine moieties of DNA patches. The Thr-NP structure showed higher lifetime with ice molecules.

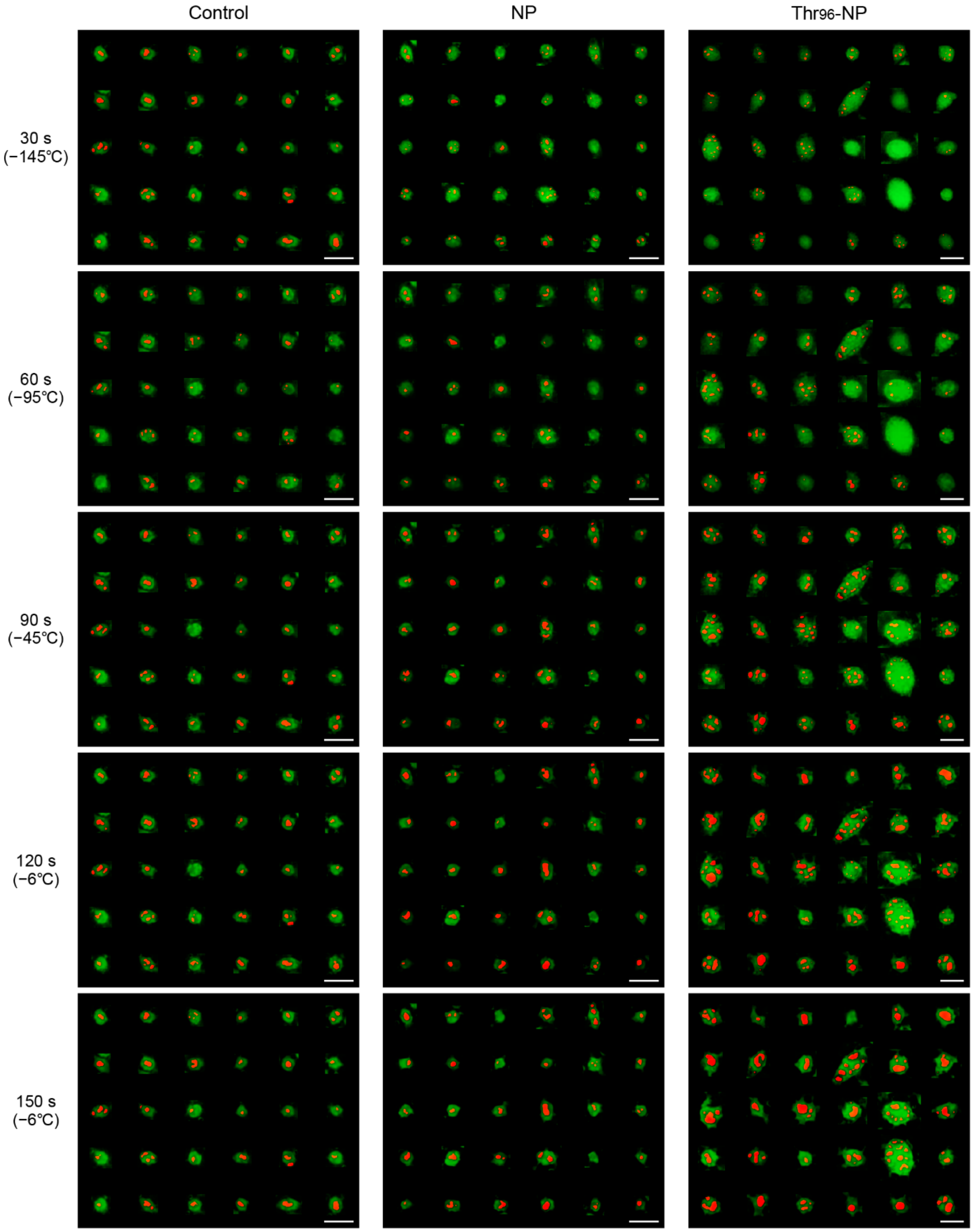

Fig. S5. Deep-learning-based intracellular ice grain analysis. Results of representative 30 cells per each case at five time steps were shown. Red-colored regions indicate the detected ice grains by the deep-learning algorithm. Scale bars: 20 μm.

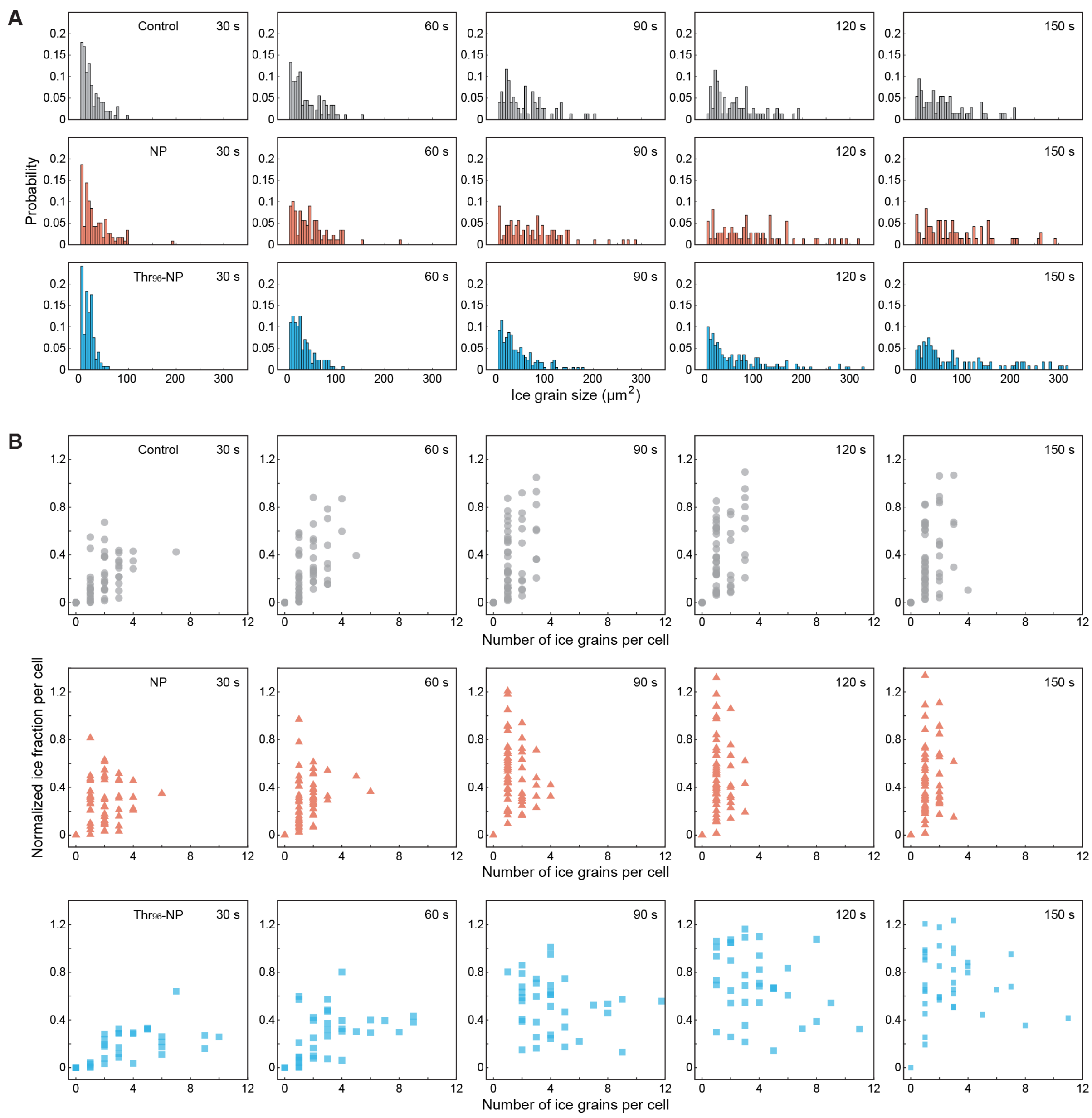

Fig. S6. Time-dependent growth of intracellular ice grains. (A) Probability histograms of the size of intracellular ice grains during the freeze-thaw process as depicted in Figure 4E. (B) Time-dependent growth of intracellular ice grains of the control cells (n = 56), NP-treated cells (n = 60) and Thr_{96}-NP-treated cells (n = 37). The area fraction of total ice grains per cell is plotted against the number of ice grains. The normalized ice fraction is calculated using the initial cell area measured at 30 s.

Table S1. Staple list of the 6HB design.

Name	Sequence (5'→3')
**Staples used for 6HB design**	
**6HB-001**	GCTTGACTCACCGCCGAAAATCCTGTTTAGTTTGGCCGTCTA
**6HB-002**	AACGAGTATATATTCAGAAGCAAAAAAACATTATGTTTTTAG
**6HB-003**	GTGAAGTTTCAAAAACCATAAATCAAAAAGACTTCCCAACAG
**6HB-004**	GCTGAACTCACCACCAGCAGAAGATAAATAAAGCAGCAAATC
**6HB-005**	GAGTAGACGAGAAGTGTTTTTATAATCAAACATCACAATATT
**6HB-006**	CAACACTATAACAACATTATTACAGGTAAGGCATAAATAGCG
**6HB-007**	GGGGCGCTAAATCAAAGCTAAATCGGTTGTACCAGGCATTAA
**6HB-008**	CCGAACTCAAAGTACAACGGAGATTTGTCAATCATCCAGGCG
**6HB-009**	ACCGATAAAATAATTTTTTCACGTTGAATTAAACAACCGATA
**6HB-010**	TCAGGAGACCCTCAAGAGAAGGATTAGGGTGTATCCCGCCAC
**6HB-011**	CCCTCAGATTAGCGTTTGCCATCTTTTCCCTCAGATTGACAG
**6HB-012**	CAATCAACCAAGACTCCTTATTACGCAGAGTTTATGGGCGAC
**6HB-013**	TTTGATATTAGAGAAGAGGAAGCCCGAAATCAGGTTGTGTAG
**6HB-014**	TTTTTGTGTTTTTATCCTGAATCTTACCAAATAAGAATAACA
**6HB-015**	ATAGATATACAGTAATAAGAGAATATAACCTGTTTCGAGCAT
**6HB-016**	ATATAACGCAAACATAGCGATAGCTTAGGCTTAGGAGAACGC
**6HB-017**	GTAGATTGATTCATCAATATAATCCTGAAATAAAGCTTTTAC
**6HB-018**	GTCAGTAGCCAGCAATTGAGGAAGGTTACTGGGGTGCGTAAG
**6HB-019**	GGCAGATCTCAAATATCAAACGCTCAATCGTCTAAAAATACC
**6HB-020**	TAAAAGAAATACTTCAACAGGAAAAACGGCTGCATCGAGCAC
**6HB-021**	GCGGTCAAGAACTCAAACTAAAGGAGCGGGCGCAAGGAAGGG
**6HB-022**	TCAGGGCAAGTTTTGCCGGCGAACGTGGCGGGCAAGTTCCGA
**6HB-023**	ATAAATAATAATGCTGTAGAGACTGGATAGCGTCATAATAGT
**6HB-024**	AGAATTAGCAATAAAGCCTCATTGCGGGATTTCAA
**6HB-025**	TTGAGATATAACGCAAGAAGTTTTGCCAAGCTGATTTAATCA
**6HB-026**	TCAGTGAATCATAACCCTCCATTACCCAAATCAGCACAAGAA
**6HB-027**	AATTGTGCCGGAACCCTTCATCAAGAGTAACAAGAGTAATGC
**6HB-028**	AGGGTAGGACCAACTTTGATCACCCTCAGCAGCTGCGCTTTT
**6HB-029**	AGGCTCCCTTGCTTGGCTTGCAGGGAGTCAGGAAGACGTTAG
**6HB-030**	AAACTACGTTGCGCCGACAATGTACCGTAACACCCAGCCCAA
**6HB-031**	CATCCAAGAGCTGAGTTTGACCATTAGAATAAAAAACCCTGT
**6HB-032**	GCTCAGTTATAAGTCCCTCATTTTCAGGATTTTTTTCAGTGC
**6HB-033**	TGAATTTGTTTAGTACCGCCCTCATTAAAGCCACCTCACAAA
**6HB-034**	CGGAACCCGCCACCTCAGACGATTGGCCGAGTAACTCGATAG
**6HB-035**	TTTGGGAAGCCGCCACCAGAAGGTGAATTATCAGCCGGAAAT
**6HB-036**	TAGAAAAACGCAAAGGGAGGGAAGGTAATCGTAACGCCCTTT
**6HB-037**	AGATAACTAGAAAATTCATAAGTCAGAGGGTAAGTCTGAACA
**6HB-038**	TCTTTCCCCATATTCGCATTAGACGGGAGCTTCTGGGTATTC
**6HB-039**	GCAAGCCTTAACGTCAAAATATTAAACCAAGTACGTCATTCC
**6HB-040**	GTAAAGTGTTCAGCATAATCGGCTGTCTTCGCTATTTCTTAC
**6HB-041**	AAGGCGTAGTCCTGAACAATTAATGGTTTGAAAAAATCTTCT
**6HB-042**	AAGAGTCAGACTACAAATATATTTTAGTTGTAAAACCTTTTT
**6HB-043**	TACCTGATATATGTAAATGAAAATCGCGCAGAGAATTGAATA
**6HB-044**	CTTCTGAATTATTTTAACGGATTCGCCTATGGTCATAATTTT
**6HB-045**	TACATTTTTCAGGTTTAACAACAACTAATAGATCATATCTTT
**6HB-046**	GGGAGCTGTGCTTTAACCTGTCGTGCCACTCATGGTTAACCG
**6HB-047**	AGCCCGAAAAATCCTTTCACCAGTGAGACGAGAAACCATCAC
**6HB-048**	GTCCACTCAGCAGGCTGGCCCTGAGAGAGCCCCCGGCACTAA
**6HB-049**	GCTCATTTTACCTTTATTCAACCGTTCTGAGGGGGACTAATG
**6HB-050**	GCAAAAGAAGGCACGAGAGTCTGGAGCAAATCTTGTCCATGT
**6HB-051**	CAACTTTATTTTCTAAAAGCCCCAAAAATAAAGGCTATCGGT
**6HB-052**	GCCCCCTAACAGTGTGTTAAATCAGCTCGATAGCAGTCGAGA
**6HB-053**	TTGCCTTGTAATCACATTAAATGTGAGCTTGATATGCCTCCC
**6HB-054**	TACCAGAAAGTAAGTGTAGATGGGCGCAATATTGAAACATAT
**6HB-055**	ACTTGCGGCGAGGCAGCTTTCCGGCACCGAATTAATACAAAA
**6HB-056**	AATTGAGAAGCCAACGGTGCGGGCCTCTTTCCTTAACAATAA
**6HB-057**	AGTGAATAACAGTACCCAGTCACGACGTTAATTTCATCATAG

**6HB-058**	CAAAGAATGAGTAATCGAATTCGTAATCGATTGCTCCTACCA
**6HB-059**	GCGTTTTGAGAATGGGGTGAGAAAGGCCGCAACTACTTAATT
**6HB-060**	TATTAGTGGCACAGATAAAGTGTAAAGCTCTAAAAGTGCCAC
**6HB-061**	TTTGCCCATTAAAGCCAACGTCAAAGGGCCGTAAAATTTAGA
**6HB-062**	AATACTTGAGCATATACAGGCAAGGCAACTATATTCGCAAAT
**6HB-063**	CTGACTAAAGATTAGTACCTTTACTAATAGTAGTACGGATTG
**6HB-064**	GAGTGAGGAAAGGAGCAAATGAAAAATCACAGAGGACATCGC
**6HB-065**	CTTTCCATGACCCTCAATCAATATCTGGGCGCTCAGGACATT
**6HB-066**	TCGGCCAGCCATTGCTTTGATTAGTAATGTGAGGCGACAGGA
**6HB-067**	AGGGTGGCGATCGGCCTTGCTGGTAATAGCGTATTGCTTAAT
**6HB-068**	TTGCCCTGGGGAAATTGGGGTCGAGGTGCGAAAAAAACAAGA
**6HB-069**	CACCATCTTCTCAACATGTTTAAATATGGAGACAACAGTTC
**6HB-070**	TGCCGGATTTGCAACAAAAGGAATTACGGAAAGATTCTACGT
**6HB-071**	CGCAAGGTACATTTTTCATTT
**6HB-072**	TCAGGTCTTGTTTACCAGACGACGATAAATCTACAGAACGAG
**6HB-073**	TGAACGGTGGCTGAGAGGCGCAGACGGTATCATCGCCAGCGA
**6HB-074**	CGGTTGACGAAGAGGACAGATGAACGGTAATCATAAGACTTT
**6HB-075**	AAGCAAATCGCTGATCGAGGTGAATTTCAATCTCCAGGAACA
**6HB-076**	TCGCATTCCATGACAACAACCATCGCCCTATTTTGTCATAGT
**6HB-077**	AGGAACGGCCACCAATAGCCCGGAATAGATTAGCGCATGAAA
**6HB-078**	CCAGCTTATCACCCTCAGAACCGCCACCTGGCCTTTTTGATG
**6HB-079**	CGGATTCAGGCAGGCTCAGAACCGCCACATAATCAGGCATTT
**6HB-080**	ATAGGTCTAAACCACCACCAGAGCCGCCTTGACCGCCATTAC
**6HB-081**	CTGCCAGCGATTGAGACACCACGGAATATATGTTAGAATACC
**6HB-082**	ATCGCACCAATGGTTTACCAGCGCCAAATCGGCCTAAGAGCA
**6HB-083**	AAACCAGAGGGAAGATTTATCCCAATCCAACGCTAAGTTGCT
**6HB-084**	TTGGGAAGGATGAAAATAGCAGCCTTTAAGGCTGCCCGCGCC
**6HB-085**	GCTGGCGCCAATCATAATGCAGAACGCGAGTACCGTAATTTA
**6HB-086**	AACGCCAATGAAAAATAATATCCCATCCCGATTAATTACTAG
**6HB-087**	CAGTGCCCTTTTTCCTTTTTAACCTCCGATTAAGAAATTAAT
**6HB-088**	CCCGGGTACCTGATGCAAATCCAATCGCGACTCTAAGAAAAC
**6HB-089**	TTCCTGTGAAACAAGCACGTAAAACAGATTGTTTGTATTCCT
**6HB-090**	TACGAGCCTGTCAGATGAATATACAGTAAATTCCAACAATTC
**6HB-091**	CCGGATAATTGCCTCAACCTAAACGAGAAACACCAAAGGCTA
**6HB-092**	CAAATAATCATCAAGTAGCGACATCATACATGGCTCCTGTAG
**6HB-093**	AAGAACGGGGCGATCGCTCAACATAGGAATCATTAGCAACTG
**6HB-094**	AGGAGCACGGAAGCACAATATTTTAGACTTTACAACACAACA
**6HB-095**	CATTAAAACAGAGAACATTAATTGCGTTTCAGTTGTCACCTT
**6HB-096**	TTATACCGGAAGTTAAAACTAGCATGTCGTACAGAAAGGGAA
**6HB-097**	TCGGTCAAAGGCCGGAACAAACGGCGGAGCCAGCAGCCACCA
**6HB-098**	GGCAGAGCTGTTTAGATGTGCTGCAAGGTAATTTAATCAACA
**6HB-099**	AGAAAACAATTCGAGCTTCCATTGAATCCCCCTTAAATCGTC
**6HB-100**	GCGCCGCGCCAGAATCCTGCTGCGCGTAACCACCAAAGTGTA
**6HB-101**	TAGCGTAGAATTGCGAATACGCCTGTAGCATTCACCCAGTAC
**6HB-102**	AGAAACAACTGGCATGATTACAAGAATTGAGTTAAATCAGAG
**6HB-103**	AAAATTATAGAATCCTTGAAAAAGAAGATGATGTTTTTCAAT
**6HB-104**	AAGAAAGTTTTTCTCTTATAAATCACACCCGCCGCGGGCGCC
**6HB-105**	TATTCGGTATTTAAAAAGTTTTGTCGTCAATAGAAAAAAAAA
**6HB-106**	CCCTGAATCCAGCCGTTTTAGCGAAGCCCAATAATCAGGAAG
**6HB-107**	GAGAAAAAAAGCTTGTTAACAATTTCATTCGCTATTCGCTGAG
**6HB-108**	CCAAATCGATGGCCTTGAGTGTTGTTCCGATGGTGCAGCTGA
**6HB-109**	TATCAAAATAATGGGAAGGAGCGGAATTACGTTATTAGCTGT
**6HB-110**	GTATAACAAACAGGCATCACGCAGAAATGGATTATTCAGAGC
**6HB-111**	TAGTAAAACGAACTAACGGAAGGCTTGCCCTGAACTGCTCAT
**6HB-112**	TAAATGACAACAGTGCCTTTAATGAAAGACAGCATTGCTAAA
**6HB-113**	ATACAGGGAGGCTGAGACTCGTTCCAGTAAGCGGACAGTCTC
**6HB-114**	TTAAGAAAGGAAACATAAAGGTGCCGTCACCGACTACAAAGT
**6HB-115**	CAATAGCACCCAGCTACAATTTTATTTTCATCGGTAGAACAA
**6HB-116**	TAATGGAAACCTTGAATTTATCATACCGACCGTGTATATGTG
**6HB-117**	GTAAAGATTCATTCATTTTTGCGGATGGGCAAACTAAATATC
**6HB-118**	CCTCAGACCATCAATGGTAATAAGTTTTTCTGAAAGGGTTTT
**6HB-119**	ATTCAACTTTGAGGAGCAATAGCTATCTAATAACGGCAAACG
**6HB-120**	TAAAAACGCAAAGCTCAGATATAGAAGGCAAGATTACGAGCG

**6HB-121**	GTAGAAAAAAGGGGGTATCATATGCGTTCAACATGACAAAAG
**6HB-122**	GCTGAATTTAAAGCGAACCAGACCGGAACTTAGAGAAGTACG
**6HB-123**	CAGATACTTAGGAATCAGGACGTTGGGATTCAACTAAATTAA
**6HB-124**	TACTTAGTCGAAATTAAAACACTCATCTAAAATACGAATCGA
**6HB-125**	TCAGAGCAGAGCCAAGACTGTAGCGCGTGAAACCAAACCCGT
**6HB-126**	GTCTGAGAATAGTGCTTCTGTAAATCGTTGAATTACGACGGC
**6HB-127**	GCTGAGATTAACACGCGCGAACTGATAGCCTGAAAGCCTAAT
**6HB-128**	CATCAAATTATAGTTTAAATGCAATGCCTTCCCAATGCTCCT
**6HB-129**	AATCGGCGATAGGGCACTACGTGTAGGGCGCTGGCAAAGAAT
**6HB-130**	CTTGAGTGCCTATTGGATAAGTGTGAGTTTCGTCAAGTTAAT
**6HB-131**	AATACGTCTTTAATCGCCTGCAATAGAGCCGTCAAGAATGGC
**6HB-132**	AACCCTCAGATTTAAAAGGTGGCATCAATTCTAATTTCTGCG
**6HB-133**	GAGGTTGTCCGTGGGAAACGTCACCAATTTTCATCAAATCAC
**6HB-134**	ATCGGGAGTGAAATAATCCTTTGCCCGAATCATCAGATTATA
**6HB-135**	TTGTAGCGTCTGTCAGGCCGATTAAAGGGCTTTGATAATGAA
**6HB-136**	TTATCAGAAAAGGATTCAGCGGAGTGAGTTTCCAGATTGTAT
**6HB-137**	CTGGCCAAATACCGAACGAACCAGTCACACGACAATTACATT
**6HB-138**	TTGTGAAATACCAGTACCACATTCCAATACTGCGGAGAACTG
**6HB-139**	CACTACGAATACACCCGCGACCTACGTAACAAAGCGAAAGAG
**6HB-140**	TTCATGAAAGCGCGAAACAACGGCTACAGAGGCTTCGGAACG
**6HB-141**	CAGCACCTAGCGTCCCACCGGAAGAATGGAAAGCGATCAAGT
**6HB-142**	TAAGAACGGAGGTTAATTTGCCATTGAGCGCTAATCCTCCCG
**6HB-143**	CAGTATAAATCGCCTCCAGACGACCGCACTCATCGAGGGCTT
**6HB-144**	AAAAAGCGCATTTTCGAGCAATAAGAATAAACAATGATAAAT
**6HB-145**	AAAAGTTACCACCAAAGGGTTAGGCGAATTATTCATGCGGAA
**6HB-146**	AACAGTTCTAACTCTAGAACCCTTCTGACCCTAAATGAGGCG
**6HB-147**	ACCGCCAACGCGCGCGCGTACTATGGTTGATTTTACACCGAG
**6HB-148**	AGAGGCTGAGGGTATGAGATGGTTTAATAGAAAAATCATCAG
**6HB-149**	CATAGGCTAATCGTTCCATTAAACGGGTTTGACCCCCTGATA
**6HB-150**	TAGGAACAAATTTTCCCGTATAACACAGACAGCCCTTAAAAT
**6HB-151**	CCAAGTTACCGAGCCATTATCATAAACAAACATCAGAGGATC
**6HB-152**	TAAACAGAGAGCCTTTGAAGCCTTAAATCTTATCCGTGCCGG
**6HB-153**	ACAACATAATTCTGATATTTAACAACGCATACAAATACGCCA
**6HB-154**	GTCAGGAAGAGGTCCATATAACAGTTGATGAGTAACTTTACC
**6HB-155**	GTATTAAAGTGTACAAATAATTCGCGTCCTCAGAAACCGTAC
**6HB-156**	CAAAAGAATGAAATGGACGACGACAGTAGACAAAATTTGTCA
**6HB-157**	CATTAGCTAGCCCCCTTATTAGAGCCAGCAAAAGATGAGCCA
**6HB-158**	GACAACTAGATGATGGCAAGGATTTAGAAGTATTTTAGATAA
**6HB-159**	TACATTTGTCGGGACCTCGTTAGCAGTAATAAAAGCTGCCCG
**6HB-160**	AAAATGTAATATGAATGCGATTTCAAATGCTTTAAGTCAAAT
**6HB-161**	GCGGGATTAATCAGGTATGGGATTTTGAGGACTAATGTACCC
**6HB-162**	TATTCATACGTTGGCAGATAGCCTCACCAGTAGCATAATGGG
**6HB-163**	GACCTAAGGGTTTTCATAAATCACCGGAATCATAAGTTGGGT
**6HB-164**	GGGTTGAACCAGGCTCGGAACCTATTATAACGGGGAACCAAT
**6HB-165**	AAAAGAATACATACCGAGGAAACGCAATTACCGAACGTGCAT
**6HB-166**	ACGGTACTACAGGGGGGAGAGGCGGTTTTCCAGAACTTGCCT
**6HB-167**	TAATAAATTGGGCTGCTATTTTTGAGAGAAACCAAGTAAGAG
**6HB-168**	ACTAAAGACGATCTATTGTAAACGTTAAACGCATAGCTTGAT
**6HB-169**	ATTTTGCAAGCAAAGCCATTCGCCATTCCAGAGAGAAACGAT
**6HB-170**	TTTCCCTATTACATCATGCCTGCAGGTCAAGACAATTGGGTT
**6HB-171**	GATTATCCGTATTATGTTATCCGCTCACACAGTACAAATTGC

Table S2. Staple list of the 12HB design.

Name	Sequence (5'→3')
**Staples used for 12HB design**	
12HB-001	GTAGCGACAGAACGTCAGAAAGCCAGTCTGAATTTA
12HB-002	GAAACGTCACCACCCCCTTGACGATTTGATACAGGA
12HB-003	CCAGCAAAATCACGGAACCCCAGAGCGCCTTGAGTA
12HB-004	CGGAAATTATTCGACAGTAGCATCTGTGTACCCCGG
12HB-005	GGGCGACATTCACGGCACCTGGGATAATTGTATAAG
12HB-006	TCAATAGAAAATGCCATTCCCGTCGGTTAAAATTCG
12HB-007	GCAAACGTAGAATGCAAGGCCCGCTTTAATGAATCG
12HB-008	AAGAACTGGCATACGACGTTAATGAGGGGCGCCAGG
12HB-009	CAGAAGGAAACCGCAGGTCAACATACCAGCTGATTG
12HB-010	AGCAATAGCTATGCCTAATCAACGCTTCGGCTGTCT
12HB-011	AATTGAGTTAAGCCCAATCTTGCTATAAGTACCGCA
12HB-012	AGAGGGTAATTGTGAAAATTCCCGACCATCGTAGGA
12HB-013	CATCGATAGCAGCACATCGGCCAAACAAAGCGTCATACATGGAGT
12HB-014	CACCATTACCATTATCTTTTCACAGGAGATAAGTTTTAACGGCTC
12HB-015	CTTGAGCCATTTGGAACCGCCGAGCCGCATAAACAGTTAATGCTG
12HB-016	GAGGGAGGGAAGGTATCGCACAGATGGGAAAAGCCCCAAAAATCG
12HB-017	GTTTACCAGCGCCAAAACCAGCAAACGGATTGTAAACGTTAAGCT
12HB-018	GGAATAAGTTTATTTTGGGAATAAATGTTGTTAAATCAGCTCTTA
12HB-019	ACTCCTTATTACGCAACGCCATTAATTGGGGAGAGGCGGTTTAAG
12HB-020	CGCAATAATAACGGCAGTGCCAAGTGTATTTCACCAGTGAGAGAA
12HB-021	AGCAGATAGCCGAACCCGGGTTATCCGCCTGGCCCTGAGAGAGAT
12HB-022	ATAAGAGCAAGAAAATAAACACAATTTTTCCAAGAACGGGTACCC
12HB-023	ATATCAGAGAGATATTTTTTGGAAGCCTCAAGCAAGCCGTTTGTT
12HB-024	AACTGAACACCCTGAGAGAATCGCGAGGGCCCAATAGCAAGCGAC
12HB-025	CCGTATCACCGGAACCGCGATGAACCCATTAAACGGGTACTAC
12HB-026	GTGTGCCGTCGTCATTTTCAATCATAACCTAAAACGAAACCTC
12HB-027	ACAGATTAGGATAACACTGACCTGCTCTTTGACCCCCAGAGGC
12HB-028	TTGAACTAGCATACTTTTGCCAGAGGTCCAATACTGCGGCATT
12HB-029	CAAAAGTCTGGATTTTTAATAAAAACTCAAATGCTTTAAAGGA
12HB-030	CATTATTTTTGATGTGTAAGTAAGAATCAAAAATCAGGTAGCT
12HB-031	GCCAGTGGACTACCATCACAAATATCATTCTGGCCAACATCTG
12HB-032	GTGGAGTGTTGAGCACTACAGCAGCTAAGAATACGTGGCGAAA
12HB-033	CCCTAAAATCCTTGACGGGGTCAGTTCTTTAATGCGCGATGGT
12HB-034	TTCCGTAGAAATAAAGCCTAACAGTCGCCTGATTGCTTTACAT
12HB-035	CTCACCTGAACTATTTAAAATTGCGGCGAATTATTCATTAAAT
12HB-036	ATCACAGCTAAGAGCCAGCTACCATAAACAAACATCAAGGTAC
12HB-037	ATAGCCACCCTGACCAACTAATGCCACTACGATCGGAACAAGG
12HB-038	AGTACCAATAGCCGGAACAAGAATACACTAAACCGCTTTGTGA
12HB-039	AGACGTACAAAATAAATTACCAAGCGCGAAACAACCGATATGA
12HB-040	ATGATATTTCAGGCTTTTATAAATATTCATTGTTGCTCCGGTG
12HB-041	ATCATTTAAATACCCTCGAGAAAACGAGAATGGGAAGCATCTG
12HB-042	ATGCAGGGTGACCAAAAGCTGACTATTATAGTCAAATATGCAA
12HB-043	AGTCTTTGGGGAAGCATCAACCCTTCTGACCTCTACATTTTTT
12HB-044	TAGCAGGGAGCCAACAGTATATTTTTGAATGGTACCGCCTCAC
12HB-045	GGTGCGTGGCGAAGATAAGCCCTAAAACATCGAACTCAAATAA
12HB-046	ATCCGGGCTTATAACGTCAAGTTACAAAATCGTTTCCCTTTTA
12HB-047	TATCAATTTAGCACGTAACCTGAGCAAAAGAAAGTGAATTGCT
12HB-048	GACAAAAGTACGAATAATAATTAATTACATTTTTACCTTCTTTTTC
12HB-049	AGATCTCCAAAAAAAAAGTGAGAATAGAACCGCCCGGAATAG
12HB-050	AGCGTTTATCAGCTTGCGTATGGGAGCAAGCCCAGGCGGAT
12HB-051	TTGATACCGATAGTTGCAAAGTTTGTCACCATCCTCAAGAG
12HB-052	TTTTTAAATATGCAACTGCATAAAAAGCCTTACGGTAATCG
12HB-053	TTAATATAACAGTTGATGCAAGGCCATATATGGTCATTGCC
12HB-054	TCATTTGACCATTAGATTCTACTAATTCAAACGGAGAGGGT
12HB-055	AAAACGCCAGAATCCTGCGTATAACAAGTTTCACTATTAAA
12HB-056	AACCCGAGTAAAAGAGTTGCGCCGACCCTAACCGAGATAGG
12HB-057	AATGCAATACTTCTTTGAGCGGTCCGGCGAAGTTCCGAAAT

12HB-058	AGCATAGGTCTGAGAGAAAAGCCTAACAGTATAATTTACGA
12HB-059	CGTGGTTATATAACTATGGCGTTAAACATGTAACAATAGAT
12HB-060	ATAAAGACAAAGAACGCCCTAAATAGAATATATAAACAACA
12HB-061	AGCCTTTAATCTTTCAAACCACCCAGAGGGTTGATATACTT
12HB-062	ATTTCTTAAAACGTTAGTGTACCGTTAGCGGGGTTTTGGGT
12HB-063	CAACAACCATTCATAGTGCCTGTAAAAGTATTAAGAGGCCC
12HB-064	TCTGGAAGTTATTAAGCGATAAAAAGCAAACAAGAGAACAG
12HB-065	CGAACGAGTAAACATCCCTGAGTAAGAGATCTACAAAGTAT
12HB-066	ATGGTCAATAAGCTGAACGGAGACTTCTAGCTGATAAAATT
12HB-067	TATAATCAGTCTATGGTGCCGTAATTCCAGTTTGGAACGCG
12HB-068	GCAAATTAACCACCACATTAGAGCCTTATAAATCAAAACGG
12HB-069	CATCACTTGCCGCTAGGAAGGGAACGAAAATCCTGTTTGTT
12HB-070	ACCTCCGGCTCCGGAATATCGCCAAAGAAAAATAATATTTA
12HB-071	GATGCAAATCTACCGACCATTTTCTGCAGAACGCGCCTTTA
12HB-072	TTTCACGAAAGGAAACCCTCATACTCAG
12HB-073	GTGTTCCAGTAATAAATCCTCATTACTGTAGCAACAACTTTGA
12HB-074	AAGTACTGGTAGTTGAGGCAGGTCAATTAGCGTAAACAATGTA
12HB-075	AAGGTGCCCGTCACCAGAACCACCAAGAGCCATTTCCAGCAGC
12HB-076	TAAATAATCAGCGCATCGTAACCGTTCGGCCTCGGTTGTACAT
12HB-077	TGAGTATTTAACGGATTGACCGTAAGCTTCTGTAGCAAATCAT
12HB-078	AGCTAAATTTTGAGCGAGTAACAACAGGCTGCTAGCATTGATT
12HB-079	GAACACGCGCGCGTTGCGCTCACTGCGATTAATCCTCGTGAAC
12HB-080	GTTGTTTTTCTAAGCCTGGGGTGCCTGTAAAAGCGCGTAGAGG
12HB-081	CGGCTCACCGCTCACAATTCCACACGACTCTAGCGTAACCGTT
12HB-082	GCATTTATCATATCCTGAATCTTACTTGCCAGATCATATCAAA
12HB-083	AAGTTCGAGAATAAATCAAGATTAGCAAATAAATAAACATAGG
12HB-084	TGTTTTACCGCCGTTTTAGCGAACCAGCAGCCTTTGAAACAAT
12HB-085	TCCAGACAAATCAGCGGTATTCTAAGAAAACATAACTTCTGAGAGA
12HB-086	TTGAGGCCTTGATATTCAATTTTCGAGCGGAGGCTCCAAGAGG
12HB-087	CAGTCGCCGCCAGCATTGATAATCAATTTTCTTTTCGAGTGCG
12HB-088	CCTGAGCCACCACCCTCATCCCTCAACGATCTGCCGACAATAT
12HB-089	GAAGGGTCACGTTGGTGTTCCAGCCCCTCAGAAAAGTACTTTT
12HB-090	TTTGATTCTCCGTGGGAAGCAAAGCCATACAGTCCCAATAACT
12HB-091	TTTTGCTTTCATCAACATGGGCGATCATCAATACATTTCCGCG
12HB-092	TATTTGAGCTAACTCACAGGGTTTTACGAGCAAGAAGTGTTGA
12HB-093	GCAAGAGCCGGAAGCATAAAGCTTGCGCTTAACTGTCCAAGCC
12HB-094	GCAGCTGTGTGAAATTGTACCGAGCCAAGTGTATTAGTAACTA
12HB-095	AACCTTTGCACCCAGCTAGCCATATACTAGAACTACCTTTAGA
12HB-096	TTTTTTGCGGGAGGTTTTTTTAACGTAAATAAATGTAAAAACC
12HB-097	AAAGGCTTTGAGGACTAGAAGTTTGGTGTACTGGCTGACCTTCAT
12HB-098	TCGAGCGAAAGACAGCAAGGCACCAAGGGAATTCATTACCCAAAT
12HB-099	TTGCAGGGAGTTAAAGGACACTCATCCATGTATAAGGCTTGCCCT
12HB-100	GTTGCGGATGGCTTAGAGGATAGCGGGGTAATTCAACTTTAATCA
12HB-101	TCCGAGAGTACCTTTAAAATCCCCCCAAAATACTGGCTCATTATA
12HB-102	TAGAAGCGAACCAGACCACCATAAGCAACACACGTTAATAAAACG
12HB-103	GGTTGGATTATTTACATAAAGGGACAAACCCAGTTGAAAGGAATT
12HB-104	CCAGCTCATGGAAATACGAAAGCGAAATGAAAGCACTAACAACTA
12HB-105	GTAATCCAGAACAATATCTATTAGATTAACAATTTGAGGATTTAG
12HB-106	ATCGATAGCTTAGATTAACGGATTACCTTTTCCGAACGTTATTAA
12HB-107	TTGCGCTATTAATTAATCGCAGAGTAGATTTTTGCGGAACAAAGA
12HB-108	CGCAATCAATATATGTGGATGATGATCAAAAATATTCCTGATTAT
12HB-109	GTAGCAACGGAAATACGTTTGAAAAATCTTGACAAG
12HB-110	GGATCGTCACGAGGCAAGAGGCGCACAAAGCTGCTC
12HB-111	TCGGTCGCTGCGATTATGTGTCGAAACACCAGAACG
12HB-112	GATAAGAGGTAATCGTCGCAAAAGTTACCTTATGCG
12HB-113	CCAACAGGTCACAGTTCTTTACCAGGACGTTGGGAA
12HB-114	TTTTAATTCGCTTTACCGAATTACGGAACAACATTA
12HB-115	CGCTCAATCGGAGATAGACCTTGCGTTATCTAAAAT
12HB-116	ATTGCAACAGACAGACAGCCACGCAGAGCCGTCAAT
12HB-117	TCGGCCTTGCACTGATAAACAGAGAGACTTTACAAA
12HB-118	ATCCTTGAAAGAATACCAGATGAAAGTTTGAGTAAC
12HB-119	TTGCTTCTGTTCAATTAAACAGAAAGAAGGAGCGGA
12HB-120	AAATTTTAATGGAAACAAAAACAAGGAAGGGTGGCAATTCATC

12HB-121	CAAGAGTGAGGACACACCCTCAGAAAGGGCGTTTTCCGTAATCA
12HB-122	CAACGTAAGACGGTCAGGGATATTTTGCTTTGCCAGCAAGGCCG
12HB-123	GACGAGAAATCCGCGAGTTTCTGTCGTCCCACCGGGAATTAGAG
12HB-124	TTGTGAAAAGTTTTGCGGGAGGCTAAATCAGGAAGAAATATTGA
12HB-125	CCAGTCAGACGACGGAACCCTAAAGAATGTGCCGGAAGACAAAA
12HB-126	AACTAACGAGGCATGGTAAAGATAGTAGGCAACTGTTGTCACAA
12HB-127	GAGGAAGTGAACCTCCCAAATCGTGCTTGTTGGGTAGTATGTTA
12HB-128	ATAGATTTGAGAGCAATCGGACTACAGGCGACGGCAATACCCAA
12HB-129	AAGTATTGTGAGGCGGAAAGCACGCTGCGAGGATCCAAAGTTAC
12HB-130	TTTTAAATATACAGAACGCTCGTTTAGTTTACAAACAATGAAAT
12HB-131	AACCACCATAAAGACAACGCCAATAAGAGAAACGAACCCACAAG
12HB-132	CAGATGATTAGAACTAATAAGTTAATGGTTTACAGAACAAAGTC
12HB-133	AACCGGATACCGAACTCAGAGCCCAGTTTCGTCATAGATGAAAC
12HB-134	ATTCAGTGATACTTAGGAACCCATAAATGAAAATCACCCAGTAG
12HB-135	AGTAGTAAATCGCCTGCTACAACTAGCGTAGAGCCGCTCACCGA
12HB-136	ATTTTAAGAAGCGAGAACGCAAGAATAAAGAGCTTTCACCGATT
12HB-137	GAAAAATCTTATCATAGCAATGCAATAAATGCCATTCTCATATG
12HB-138	TTACAGGTACATAACGGAAAGGCAAGGTGGCGGTGCGACACCAC
12HB-139	ATCTTTAGGAAATCTATCGAGGTTGCTTTGCCCAGTCGATTAAG
12HB-140	AGATAATACCCGCCTGCCCCGATCCCGCCGCATGCCTGAGGAAA
12HB-141	CAATTCGACCCAGCAGAGAAAGGGCGCTGGTCGAATTAAAAGTA
12HB-142	ATTATCATTTCAGGTTATTGAGACATAATTTATTTATCCCAATA
12HB-143	ATTATCATCTTATTTGGCAGAGGCGTGTGATCAAAAAAGCGCTA
12HB-144	AATATAATCTACTTCTCGACAAAATTTCATAAACAGGGAGAATT
12HB-145	GAATTATCACCGCACCCTCAGAACGGACAGCCCCGAATATA
12HB-146	ATGCTGTAGCTCAACCAAAAACATTAGGGACGACATTAAAGGT
12HB-147	AACCTATTATACCAGTTTGACGCCACCCTCAGCCTATTTCGG
12HB-148	GAAACATGGCATTCCATAGATTTGTATCATTGGGCTTGAGA
12HB-149	TGGTTTAATTAGTAAAACATGACCCTGTAATGTCAATCATTCT
12HB-150	AATTGCTGCCCACGCATAAAGTACAACGGGTTTAGACTGCTT
12HB-151	ATAAAAGAAACGCAAAGGGCCTCTGTAGCCAAACCAATAG
12HB-152	GAACGTCAACCGAGTCAAAAACTAATAAAGCGGATTGCATC
12HB-153	AAAACAGTAATACCACATTCTCACCATTCATTTGTACGCCAACAT
12HB-154	TGCAATACATACATAAAGGTGGCAGCTGGCGCTATTCGCGTC
12HB-155	TGGCCTTCCTTCGCTATGGGCGCGACCTGTTAGCCCGAAAG
12HB-156	ACTTCAGAAGCGCAGATAGAAAGATTCATCAGTTGAGATT
12HB-157	TAGGCAGTTGGCAAATCAACTCAATCAGGCCCACAGAGCGGGATG
12HB-158	CGTCATATGATATCCATCAAAAATAGTCGTGCCACGAAAAAC
12HB-159	AGGGGCTGCATTCCAGTCGGGAAACAAAGGGGGAGCTAAA
12HB-160	CAGGTTCATATCAGGGCGATATATCTGCACGACCAGTAATATGGC
12HB-161	TAAATTCACCAGTAGATTAAGAGGATAGCTATATAGGCCGAT
12HB-162	AGAGGGATTTTAGACAGTAGAATCTACGTGACCAACGTCAA
12HB-163	GCCCTTTTTAAGCGTAATCATGGTCTGAGCGGGCTGAGTCA
12HB-164	ATAGTGAATTTATGCGTTATACAAAAGTTCCAGACTTACCGAA
12HB-165	ACGCTGGTTAAAACGAGCGTCATAGCTGTTTCCAAGCGGTCC
12HB-166	CCAGCAGGGAAAGCGGACCGAACGAACCAAACTCGTATTAA
12HB-167	ATCCTTTGCACATCGGAATTCTTACCAGTACCAATCAATTGCC
12HB-168	GCTGAGAAGAGTAGAAGCCATTAAAAATAGAAACAATAAGAC

Table S3. Staple list of the bare DNA nanopatch design.

Name	Sequence (5'→3')
**Staples used for Nanopatch design**	
**Patch-B01**	ACGTTAGTAAATGAATTTTCTGTAAGCGGAGT
**Patch-B02**	GAGAATAGCTTTTGCGGGATCGTCGGGTAGCA
**Patch-B03**	ACGGCTACTTACTTAGCCGGAACGCTGACCAA
**Patch-B04**	CTTTGAAAAGAACTGGCTCATTATTTAATAAA
**Patch-B05**	ACGAACTAGCGTCCAATACTGCGGAATGCTTT
**Patch-B06**	AAACAGTTGATGGCTTAGAGCTTATTTAAATA
**Patch-B07**	TGCAACTAAGCAATAAAGCCTCAGTTATGACC
**Patch-B08**	CTGTAATATTGCCTGAGAGTCTGGAAAACTAG
**Patch-B09**	CATGTCAAGATTCTCCGTGGGAACCGTTGGTG
**Patch-B10**	TAGATGGGGGGTAACGCCAGGGTTGTGCCAAG
**Patch-B11**	CTTGCATGCATTAATGAATCGGCCCGCCAGGG
**Patch-B12**	TGGTTTTTAACGTCAAAGGGCGAAGAACCATC
**Patch-C01**	AAAGGCCGAAAGGAACAACTAAAGCTTTCCAG
**Patch-C02**	GCTCCATGAGAGGCTTTGAGGACTAGGGAGTT
**Patch-C03**	CGATTTTAGAGGACAGATGAACGGCGCGACCT
**Patch-C04**	ACTGGATAACGGAACAACATTATTACCTTATG
**Patch-C05**	TTTTTGCGCAGAAAACGAGAATGAATGTTTAG
**Patch-C06**	CAAAATTAAAGTACGGTGTCTGGAAGAGGTCA
**Patch-C07**	TCAGGTCACTTTTGCGGGAGAAGCAGAATTAG
**Patch-C08**	ACCCGTCGTCATATGTACCCCGGTAAAGGCTA
**Patch-C09**	ATTAAGTTCGCATCGTAACCGTGCGAGTAACA
**Patch-C10**	GCCAGCTGCCTGCAGGTCGACTCTGCAAGGCG
**Patch-C11**	TGGACTCCCTTTTCACCAGTGAGACCTGTCGT
**Patch-C12**	ACCCAAATCAAGTTTTTTGGGGTCAAAGAACG
**Patch-D01**	CGTAACGATCTAAAGTTTTGTCGTGAATTGCG
**Patch-D02**	AATAATAAGGTCGCTGAGGCTTGCAAAGACTT
**Patch-D03**	TTTCATGAAAATTGTGTCGAAATCTGTACAGA
**Patch-D04**	CCAGGCGCTTAATCATTGTGAATTACAGGTAG
**Patch-D05**	AAAGATTCAGGGGGTAATAGTAAACCATAAAT
**Patch-D06**	CAAAAATCATTGCTCCTTTTGATAAGTTTCAT
**Patch-D07**	TCCATATACATACAGGCAAGGCAACTTTATTT
**Patch-D08**	CAACGCAATTTTTGAGAGATCTACTGATAATC
**Patch-D09**	AGAAAAGCAACATTAAATGTGAGCATCTGCCA
**Patch-D10**	GTTTGAGGGAAAGGGGGATGTGCTAGAGGATC
**Patch-D11**	CCCGGGTACTTTCCAGTCGGGAAACGGGCAAC
**Patch-D12**	AGCTGATTACAAGAGTCCACTATTGAGGTGCC
**Patch-E01**	ATATATTCTTTTTTCACGTTGAAAATAGTTAG
**Patch-E02**	CGCCTGATGGAAGTTTCCATTAAACATAACCG
**Patch-E03**	TTTCAACTATAGGCTGGCTGACCTTGTATCAT
**Patch-E04**	TTTGCCAGATCAGTTGAGATTTAGTGGTTTAA
**Patch-E05**	TACCTTTAAGGTCTTTACCCTGACAAAGAAGT
**Patch-E06**	CAATAAATACAGTTGATTCCCAATTTAGAGAG
**Patch-E07**	GGTAGCTAGGATAAAAATTTTTAGTTAACATC
**Patch-E08**	CTTTCATCCCCAAAAACAGGAAGACCGGAGAG
**Patch-E09**	CAGCTGGCGGACGACGACAGTATCGTAGCCAG
**Patch-E10**	ACTGCCCGCCGAGCTCGAATTCGTTATTACGC
**Patch-E11**	AGTTTGGAGCCCTTCACCGCCTGGTTGCGCTC
**Patch-E12**	GTAAAGCACTAAATCGGAACCCTAGTTGTTCC
**Patch-F01**	TGTAGCATTCCACAGACAGCCCTCATCTCCAA
**Patch-F02**	AAAAAAGGACAACCATCGCCCACGCGGGTAAA
**Patch-F03**	ATACGTAAAAGTACAACGGAGATTTCATCAAG
**Patch-F04**	AGTAATCTTAAATTGGGCTTGAGAGAATACCA
**Patch-F05**	CATTCAACGCGAGAGGCTTTTGCATATTATAG
**Patch-F06**	TCAGAAGCCTCCAACAGGTCAGGATCTGCGAA
**Patch-F07**	CGAGTAGAACTAATAGTAGTAGCAAACCCTCA
**Patch-F08**	TATATTTTAGCTGATAAATTAATGTTGTATAA
**Patch-F09**	GCAAATATCGCGTCTGGCCTTCCTGGCCTCAG

**Patch-F10**	GAAGATCGGTGCGGGCCTCTTCGCAATCATGG
**Patch-F11**	TCATAGCTACTCACATTAATTGCGCCCTGAGA
**Patch-F12**	GAGTTGCACGAGATAGGGTTGAGTAAGGGAGC
**Patch-G01**	CAATGACACTCCAAAAGGAGCCTTACAACGCC
**Patch-G02**	GCGAAACATGCCACTACGAAGGCATGCGCCGA
**Patch-G03**	ACGAGTAGTGACAAGAACCGGATATACCAAGC
**Patch-G04**	CCAAAATATAATGCAGATACATAAACACCAGA
**Patch-G05**	GAAGCAAAAAAGCGGATTGCATCAGATAAAAA
**Patch-G06**	TCAATTCTTTTAGTTTGACCATTACCAGACCG
**Patch-G07**	ACCGTTCTAAATGCAATGCCTGAGAGGTGGCA
**Patch-G08**	AAATAATTTTAAATTGTAAACGTTGATATTCA
**Patch-G09**	GGCGATCGCACTCCAGCCAGCTTTGCCATCAA
**Patch-G10**	GTGAGCTAGTTTCCTGTGTGAAATTTGGGAAG
**Patch-G11**	GAATAGCCGCAAGCGGTCCACGCTCCTAATGA
**Patch-G12**	CCCCGATTTAGAGCTTGACGGGGAAATCAAAA
**Patch-H01**	TGAGTTTCGTCACCAGTACAAACTTAATTGTA
**Patch-H02**	TCGGTTTAGCTTGATACCGATAGTCCAACCTA
**Patch-H03**	AAACGAAATGACCCCCAGCGATTATTCATTAC
**Patch-H04**	CCAAATCACTTGCCCTGACGAGAACGCCAAAA
**Patch-H05**	GGAATTACTCGTTTACCAGACGACAAAAGATT
**Patch-H06**	AAGAGGAACGAGCTTCAAAGCGAAGATACATT
**Patch-H07**	TCGCAAATGGGGCGCGAGCTGAAATAATGTGT
**Patch-H08**	AGGTAAAGAAATCACCATCAATATAATATTTT
**Patch-H09**	GTTAAAATTTTAACCAATAGGAACCCGGCACC
**Patch-H10**	GCTTCTGGTCAGGCTGCGCAACTGTGTTATCC
**Patch-H11**	GCTCACAATGTAAAGCCTGGGGTGGGTTTGCC
**Patch-H12**	CCAGCAGGGGCAAAATCCCTTATAAAGCCGGC
**Patch-I01**	CTTAAACATCAGCTTGCTTTCGAGAAACAGTT
**Patch-I02**	CTCATCTTGAGGCAAAAGAATACACTCCCTCA
**Patch-I03**	GAATAAGGACGTAACAAAGCTGCTGACGGAAA
**Patch-I04**	CATAACCCGAGGCATAGTAAGAGCTTTTTAAG
**Patch-I05**	TTTTAATTGCCCGAAAGACTTCAATTCCAGAG
**Patch-I06**	TTTCATTTGGTCAATAACCTGTTTAATCAATA
**Patch-I07**	AGACAGTCATTCAAAAGGGTGAGATATCATAT
**Patch-I08**	GCTCATTTTCGCATTAAATTTTTGAGCTTAGA
**Patch-I09**	TTCGCCATTGCCGGAAACCAGGCAAACAGTAC
**Patch-I10**	GCATAAAGTTCCACACAACATACGAAACAATT
**Patch-I11**	CCGAAATCCGAAAATCCTGTTTGAAATACCGA
**Patch-I12**	GAACGTGGCGAGAAAGGAAGGGAACAAACTAT
**Patch-J01**	CAAGCCCAATAGGAACCCATGTACCGTAACAC
**Patch-J02**	AATGCCCCGTAACAGTGCCCGTATGTGAATTT
**Patch-J03**	GAGCCGCCCCACCACCGGAACCGCCTAAAACA
**Patch-J04**	TTATTCATAGGGAAGGTAAATATTCATTCAGT
**Patch-J05**	AAAAGTAATATCTTACCGAAGCCCAACACTAT
**Patch-J06**	CCTAATTTACGCTAACGAGCGTCTATATCGCG
**Patch-J07**	ATCGGCTGCGAGCATGTAGAAACCAGCTATAT
**Patch-J08**	GCGTTATAGAAAAAGCCTGTTTAGAAGGCCGG
**Patch-J09**	TTAAGACGTTGAAAACATAGCGATTTAAATCA
**Patch-J10**	CTTTTACACAGATGAATATACAGTAAGCGCCA
**Patch-J11**	CGACAACTAAGTATTAGACTTTACAGCCGGAA
**Patch-J12**	ACGAACCAAAACATCGCCATTAAATGGTGGTT
**Patch-K01**	TGCCTTGACTGCCTATTTCGGAACAGGGATAG
**Patch-K02**	AACCAGAGACCCTCAGAACCGCCAGGGGTCAG
**Patch-K03**	ATTGAGGGTAAAGGTGAATTATCAATCACCGG
**Patch-K04**	GCAATAGCGCAGATAGCCGAACAATTCAACCG
**Patch-K05**	TCTTACCAGCCAGTTACAAAATAAATGAAATA
**Patch-K06**	CTAATTTATCTTTCCTTATCATTCATCCTGAA
**Patch-K07**	AATTACTACAAATTCTTACCAGTAATCCCATC
**Patch-K08**	TAGAATCCCTGAGAAGAGTCAATAGGAATCAT
**Patch-K09**	TTTAACGTTCGGGAGAAACAATAATTTTCCCT
**Patch-K10**	GGATTTAGCGTATTAAATCCTTTGTTTTCAGG
**Patch-K11**	TAGCCCTACCAGCAGAAGATAAAAACATTTGA
**Patch-K12**	CGGCCTTGCTGGTAATATCCAGAACGAACTGA

**Patch-L01**	CTCAGAGCCACCACCCTCATTTTCCTATTATT
**Patch-L02**	CTGAAACAGGTAATAAGTTTTAACCCCTCAGA
**Patch-L03**	GCCACCACTCTTTTCATAATCAAACCGTCACC
**Patch-L04**	GACTTGAGAGACAAAAGGGCGACAAGTTACCA
**Patch-L05**	GAAGGAAAATAAGAGCAAGAAACAACAGCCAT
**Patch-L06**	ATTATTTAACCCAGCTACAATTTTCAAGAACG
**Patch-L07**	GGTATTAAGAACAAGAAAAATAATTAAAGCCA
**Patch-L08**	ACGCTCAAAATAAGAATAAACACCGTGAATTT
**Patch-L09**	ATCAAAATCGTCGCTATTAATTAACGGATTCG
**Patch-L10**	CCTGATTGAAAGAAATTGCGTAGACCCGAACG
**Patch-L11**	TTATTAATGCCGTCAATAGATAATCAGAGGTG
**Patch-L12**	AGGCGGTCATTAGTCTTTAATGCGCAATATTA
**Patch-M01**	AGTGTACTTGAAAGTATTAAGAGGCCGCCACC
**Patch-M02**	GTTTGCCACCTCAGAGCCGCCACCGATACAGG
**Patch-M03**	AGCGCCAACCATTTGGGAATTAGATTATTAGC
**Patch-M04**	GCCCAATACCGAGGAAACGCAATAGGTTTACC
**Patch-M05**	TATTTTGCTCCCAATCCAAATAAGTGAGTTAA
**Patch-M06**	TAAGTCCTACCAAGTACCGCACTCTTAGTTGC
**Patch-M07**	AGGCGTTACAGTAGGGCTTAATTGACAATAGA
**Patch-M08**	CTGTAAATCATAGGTCTGAGAGACGATAAATA
**Patch-M09**	ACAGAAATCTTTGAATACCAAGTTCCTTGCTT
**Patch-M10**	AGATTAGATTTAAAAGTTTGAGTACACGTAAA
**Patch-M11**	GAATGGCTAGTATTAACACCGCCTCAACTAAT
**Patch-M12**	CCGCCAGCCATTGCAACAGGAAAAATATTTTT
**Patch-N01**	CCCTCAGAACCGCCACCCTCAGAACTGAGACT
**Patch-N02**	CCTCAAGAATACATGGCTTTTGATAGAACCAC
**Patch-N03**	CACCAGAGTTCGGTCATAGCCCCCGCCAGCAA
**Patch-N04**	AATCACCAAATAGAAAATTCATATATAACGGA
**Patch-N05**	ATACCCAAGATAACCCACAAGAATAAACGATT
**Patch-N06**	TTTTGTTTAAGCCTTAAATCAAGAATCGAGAA
**Patch-N07**	CAAGCAAGACGCGCCTGTTTATCAAGAATCGC
**Patch-N08**	CATATTTAGAAATACCGACCGTGTTACCTTTT
**Patch-N09**	TAACCTCCATATGTGAGTGAATAAACAAAATC
**Patch-N10**	GCGCAGAGATATCAAAATTATTTGACATTATC
**Patch-N11**	ATTTTGCGTCTTTAGGAGCACTAAGCAACAGT
**Patch-N12**	GCCACGCTATACGTGGCACAGACAACGCTCAT
**Patch-O01**	TAAGCGTCGAAGGATTAGGATTAGTACCGCCA
**Patch-O02**	TCGGCATTCCGCCGCCAGCATTGACGTTCCAG
**Patch-O03**	TCACAATCGTAGCACCATTACCATCGTTTTCA
**Patch-O04**	ATCAGAGAAAGAACTGGCATGATTTTATTTTG
**Patch-O05**	AGGTTTTGAACGTCAAAAATGAAAGCGCTAAT
**Patch-O06**	AATGCAGACCGTTTTTATTTTCATCTTGCGGG
**Patch-O07**	AATGGTTTACAACGCCAACATGTAGTTCAGCT
**Patch-O08**	AAATCAATGGCTTAGGTTGGGTTACTAAATTT
**Patch-O09**	AACCTACCGCGAATTATTCATTTCCAGTACAT
**Patch-O10**	CTAAAATAGAACAAAGAAACCACCAGGGTTAG
**Patch-O11**	GCGTAAGAGAGAGCCAGCAGCAAAAAGGTTAT
**Patch-O12**	GGAAATACCTACATTTTGACGCTCACCTGAAA
**Patch-P01**	TATCACCGTACTCAGGAGGTTTAGCGGGGTTT
**Patch-P02**	TGCTCAGTCAGTCTCTGAATTTACCAGGAGGT
**Patch-P03**	TGAGGCAGGCGTCAGACTGTAGCGTAGCAAGG
**Patch-P04**	CCGGAAACACACCACGGAATAAGTAAGACTCC
**Patch-P05**	TTATTACGGTCAGAGGGTAATTGAATAGCAGC
**Patch-P06**	CTTTACAGTTAGCGAACCTCCCGACGTAGGAA
**Patch-P07**	TCATTACCCGACAATAAACAACATATTTAGGC
**Patch-P08**	AGAGGCATAATTTCATCTTCTGACTATAACTA
**Patch-P09**	TATGTAAACCTTTTTTAATGGAAAAATTACCT
**Patch-P10**	GAGCAAAAACTTCTGAATAATGGAAGAAGGAG
**Patch-P11**	CGGAATTATTGAAAGGAATTGAGGTGAAAAAT
**Patch-P12**	CTAAAGCAAGATAGAACCCTTCTGAATCGTCT
**Patch-Q01**	GGAAAGCGACCAGGCGGATAAGTGAATAGGTG
**Patch-Q02**	TGCCTTTAGTCAGACGATTGGCCTGCCAGAAT
**Patch-Q03**	ACGCAAAGGTCACCAATGAAACCAATCAAGTT

**Patch-Q04**	TGAACAAACAGTATGTTAGCAAACTAAAAGAA
**Patch-Q05**	GAGGCGTTAGAGAATAACATAAAAGAACACCC
**Patch-Q06**	CCAGACGAGCGCCCAATAGCAAGCAAGAACGC
**Patch-Q07**	TTTTAGTTTTTCGAGCCAGTAATAAATTCTGT
**Patch-Q08**	TTGAATTATGCTGATGCAAATCCACAAATATA
**Patch-Q09**	TGGATTATGAAGATGATGAAACAAAATTTCAT
**Patch-Q10**	ATCAACAGTCATCATATTCCTGATTGATTGTT
**Patch-Q11**	GCCAACAGTCACCTTGCTGAACCTGTTGGCAA
**Patch-Q12**	GAAATGGATTATTTACATTGGCAGACATTCTG

Table S4. Staple list of the DNA nanopatch with 48 and 96 handles, and PNA probe strands with alanine and threonine moieties.

Name	Sequence (5'→3')
**Staples used for Nanopatch with handles design**	
**Patch-B02h-20nt**	CCTCTCACCCACCATTCATCGAGAATAGCTTTTGCGGGATCGTCGGGTAGCA
**Patch-B04h-20nt**	CCTCTCACCCACCATTCATCCTTTGAAAAGAACTGGCTCATTATTTAATAAA
**Patch-B06h-20nt**	CCTCTCACCCACCATTCATCAAACAGTTGATGGCTTAGAGCTTATTTAAATA
**Patch-B08h-20nt**	CCTCTCACCCACCATTCATCCTGTAATATTGCCTGAGAGTCTGGAAAACTAG
**Patch-B10h-20nt**	CCTCTCACCCACCATTCATCTAGATGGGGGGTAACGCCAGGGTTGTGCCAAG
**Patch-B12h-20nt**	CCTCTCACCCACCATTCATCTGGTTTTTAACGTCAAAGGGCGAAGAACCATC
**Patch-C01h-20nt**	CCTCTCACCCACCATTCATCAAAGGCCGAAAGGAACAACTAAAGCTTTCCAG
**Patch-C03h-20nt**	CCTCTCACCCACCATTCATCCGATTTTAGAGGACAGATGAACGGCGCGACCT
**Patch-C05h-20nt**	CCTCTCACCCACCATTCATCTTTTTGCGCAGAAAACGAGAATGAATGTTTAG
**Patch-C07h-20nt**	CCTCTCACCCACCATTCATCTCAGGTCACTTTTGCGGGAGAAGCAGAATTAG
**Patch-C09h-20nt**	CCTCTCACCCACCATTCATCATTAAGTTCGCATCGTAACCGTGCGAGTAACA
**Patch-C11h-20nt**	CCTCTCACCCACCATTCATCTGGACTCCCTTTTCACCAGTGAGACCTGTCGT
**Patch-D02h-20nt**	CCTCTCACCCACCATTCATCAATAATAAGGTCGCTGAGGCTTGCAAAGACTT
**Patch-D04h-20nt**	CCTCTCACCCACCATTCATCCCAGGCGCTTAATCATTGTGAATTACAGGTAG
**Patch-D06h-20nt**	CCTCTCACCCACCATTCATCCAAAAATCATTGCTCCTTTTGATAAGTTTCAT
**Patch-D08h-20nt**	CCTCTCACCCACCATTCATCCAACGCAATTTTTGAGAGATCTACTGATAATC
**Patch-D10h-20nt**	CCTCTCACCCACCATTCATCGTTTGAGGGAAAGGGGGATGTGCTAGAGGATC
**Patch-D12h-20nt**	CCTCTCACCCACCATTCATCAGCTGATTACAAGAGTCCACTATTGAGGTGCC
**Patch-E01h-20nt**	CCTCTCACCCACCATTCATCATATATTCTTTTTCACGTTGAAAATAGTTAG
**Patch-E03h-20nt**	CCTCTCACCCACCATTCATCTTTCAACTATAGGCTGGCTGACCTTGTATCAT
**Patch-E05h-20nt**	CCTCTCACCCACCATTCATCTACCTTTAAGGTCTTTACCCTGACAAAGAAGT
**Patch-E07h-20nt**	CCTCTCACCCACCATTCATCGGTAGCTAGGATAAAAATTTTTAGTTAACATC
**Patch-E09h-20nt**	CCTCTCACCCACCATTCATCCAGCTGGCGGACGACGACAGTATCGTAGCCAG
**Patch-E11h-20nt**	CCTCTCACCCACCATTCATCAGTTTGGAGCCCTTCACCGCCTGGTTGCGCTC
**Patch-F02h-20nt**	CCTCTCACCCACCATTCATCAAAAAAGGACAACCATCGCCCACGCGGGTAAA
**Patch-F04h-20nt**	CCTCTCACCCACCATTCATCAGTAATCTTAAATTGGGCTTGAGAGAATACCA
**Patch-F06h-20nt**	CCTCTCACCCACCATTCATCTCAGAAGCCTCCAACAGGTCAGGATCTGCGAA
**Patch-F08h-20nt**	CCTCTCACCCACCATTCATCTATATTTTAGCTGATAAATTAATGTTGTATAA
**Patch-F10h-20nt**	CCTCTCACCCACCATTCATCGAAGATCGGTGCGGGCCTCTTCGCAATCATGG
**Patch-F12h-20nt**	CCTCTCACCCACCATTCATCGAGTTGCACGAGATAGGGTTGAGTAAGGGAGC
**Patch-G01h-20nt**	CCTCTCACCCACCATTCATCCAATGACACTCCAAAAGGAGCCTTACAACGCC
**Patch-G03h-20nt**	CCTCTCACCCACCATTCATCACGAGTAGTGACAAGAACCGGATATACCAAGC
**Patch-G05h-20nt**	CCTCTCACCCACCATTCATCGAAGCAAAAAAGCGGATTGCATCAGATAAAAA
**Patch-G07h-20nt**	CCTCTCACCCACCATTCATCACCGTTCTAAATGCAATGCCTGAGAGGTGGCA
**Patch-G09h-20nt**	CCTCTCACCCACCATTCATCGGCGATCGCACTCCAGCCAGCTTTGCCATCAA
**Patch-G11h-20nt**	CCTCTCACCCACCATTCATCGAATAGCCGCAAGCGGTCCACGCTCCTAATGA
**Patch-H02h-20nt**	CCTCTCACCCACCATTCATCTCGGTTTAGCTTGATACCGATAGTCCAACCTA
**Patch-H04h-20nt**	CCTCTCACCCACCATTCATCCCAAATCACTTGCCCTGACGAGAACGCCAAAA
**Patch-H06h-20nt**	CCTCTCACCCACCATTCATCAAGAGGAACGAGCTTCAAAGCGAAGATACATT
**Patch-H08h-20nt**	CCTCTCACCCACCATTCATCAGGTAAAGAAATCACCATCAATATAATATTTT
**Patch-H10h-20nt**	CCTCTCACCCACCATTCATCGCTTCTGGTCAGGCTGCGCAACTGTGTTATCC
**Patch-H12h-20nt**	CCTCTCACCCACCATTCATCCCAGCAGGGGCAAAATCCCTTATAAAGCCGGC
**Patch-I01h-20nt**	CCTCTCACCCACCATTCATCCTTAAACATCAGCTTGCTTTCGAGAAACAGTT
**Patch-I03h-20nt**	CCTCTCACCCACCATTCATCGAATAAGGACGTAACAAAGCTGCTGACGGAAA
**Patch-I05h-20nt**	CCTCTCACCCACCATTCATCTTTTAATTGCCCGAAAGACTTCAATTCCAGAG
**Patch-I07h-20nt**	CCTCTCACCCACCATTCATCAGACAGTCATTCAAAAGGGTGAGATATCATAT
**Patch-I09h-20nt**	CCTCTCACCCACCATTCATCTTCGCCATTGCCGGAAACCAGGCAAACAGTAC
**Patch-I11h-20nt**	CCTCTCACCCACCATTCATCCCGAAATCCGAAAATCCTGTTTGAAATACCGA
**Patch-J02h-20nt**	CCTCTCACCCACCATTCATCAATGCCCCGTAACAGTGCCCGTATGTGAATTT
**Patch-J04h-20nt**	CCTCTCACCCACCATTCATCTTATTCATAGGGAAGGTAAATATTCATTCAGT
**Patch-J06h-20nt**	CCTCTCACCCACCATTCATCCCTAATTTACGCTAACGAGCGTCTATATCGCG
**Patch-J08h-20nt**	CCTCTCACCCACCATTCATCGCGTTATAGAAAAAGCCTGTTTAGAAGGCCGG
**Patch-J10h-20nt**	CCTCTCACCCACCATTCATCCTTTTACACAGATGAATATACAGTAAGCGCCA
**Patch-J12h-20nt**	CCTCTCACCCACCATTCATCACGAACCAAAACATCGCCATTAAATGGTGGTT
**Patch-K01h-20nt**	CCTCTCACCCACCATTCATCTGCCTTGACTGCCTATTTCGGAACAGGGATAG
**Patch-K03h-20nt**	CCTCTCACCCACCATTCATCATTGAGGGTAAAGGTGAATTATCAATCACCGG

**Patch-K05h-20nt**	CCTCTCACCCACCATTCATCTCTTACCAGCCAGTTACAAAATAAATGAAATA
**Patch-K07h-20nt**	CCTCTCACCCACCATTCATCAATTACTACAAATTCTTACCAGTAATCCCATC
**Patch-K09h-20nt**	CCTCTCACCCACCATTCATCTTTAACGTTCGGGAGAAACAATAATTTTCCCT
**Patch-K11h-20nt**	CCTCTCACCCACCATTCATCTAGCCCTACCAGCAGAAGATAAAAACATTTGA
**Patch-L02h-20nt**	CCTCTCACCCACCATTCATCCTGAAACAGGTAATAAGTTTTAACCCCTCAGA
**Patch-L04h-20nt**	CCTCTCACCCACCATTCATCGACTTGAGAGACAAAAGGGCGACAAGTTACCA
**Patch-L06h-20nt**	CCTCTCACCCACCATTCATCATTATTTAACCCAGCTACAATTTTCAAGAACG
**Patch-L08h-20nt**	CCTCTCACCCACCATTCATCACGCTCAAAATAAGAATAAACACCGTGAATTT
**Patch-L10h-20nt**	CCTCTCACCCACCATTCATCCCTGATTGAAAGAAATTGCGTAGACCCGAACG
**Patch-L12h-20nt**	CCTCTCACCCACCATTCATCAGGCGGTCATTAGTCTTTAATGCGCAATATTA
**Patch-M01h-20nt**	CCTCTCACCCACCATTCATCAGTGTACTTGAAAGTATTAAGAGGCCGCCACC
**Patch-M03h-20nt**	CCTCTCACCCACCATTCATCAGCGCCAACCATTTGGGAATTAGATTATTAGC
**Patch-M05h-20nt**	CCTCTCACCCACCATTCATCTATTTTGCTCCCAATCCAAATAAGTGAGTTAA
**Patch-M07h-20nt**	CCTCTCACCCACCATTCATCAGGCGTTACAGTAGGGCTTAATTGACAATAGA
**Patch-M09h-20nt**	CCTCTCACCCACCATTCATCACAGAAATCTTTGAATACCAAGTTCCTTGCTT
**Patch-M11h-20nt**	CCTCTCACCCACCATTCATCGAATGGCTAGTATTAACACCGCCTCAACTAAT
**Patch-N02h-20nt**	CCTCTCACCCACCATTCATCCCTCAAGAATACATGGCTTTTGATAGAACCAC
**Patch-N04h-20nt**	CCTCTCACCCACCATTCATCAATCACCAAATAGAAAATTCATATATAACGGA
**Patch-N06h-20nt**	CCTCTCACCCACCATTCATCTTTTGTTTAAGCCTTAAATCAAGAATCGAGAA
**Patch-N08h-20nt**	CCTCTCACCCACCATTCATCCATATTTAGAAATACCGACCGTGTTACCTTTT
**Patch-N10h-20nt**	CCTCTCACCCACCATTCATCGCGCAGAGATATCAAAATTATTTGACATTATC
**Patch-N12h-20nt**	CCTCTCACCCACCATTCATCGCCACGCTATACGTGGCACAGACAACGCTCAT
**Patch-O01h-20nt**	CCTCTCACCCACCATTCATCTAAGCGTCGAAGGATTAGGATTAGTACCGCCA
**Patch-O03h-20nt**	CCTCTCACCCACCATTCATCTCACAATCGTAGCACCATTACCATCGTTTTCA
**Patch-O05h-20nt**	CCTCTCACCCACCATTCATCAGGTTTTGAACGTCAAAAATGAAAGCGCTAAT
**Patch-O07h-20nt**	CCTCTCACCCACCATTCATCAATGGTTTACAACGCCAACATGTAGTTCAGCT
**Patch-O09h-20nt**	CCTCTCACCCACCATTCATCAACCTACCGCGAATTATTCATTTCCAGTACAT
**Patch-O11h-20nt**	CCTCTCACCCACCATTCATCGCGTAAGAGAGAGCCAGCAGCAAAAAGGTTAT
**Patch-P02h-20nt**	CCTCTCACCCACCATTCATCTGCTCAGTCAGTCTCTGAATTTACCAGGAGGT
**Patch-P04h-20nt**	CCTCTCACCCACCATTCATCCCGGAAACACACCACGGAATAAGTAAGACTCC
**Patch-P06h-20nt**	CCTCTCACCCACCATTCATCCTTTACAGTTAGCGAACCTCCCGACGTAGGAA
**Patch-P08h-20nt**	CCTCTCACCCACCATTCATCAGAGGCATAATTTCATCTTCTGACTATAACTA
**Patch-P10h-20nt**	CCTCTCACCCACCATTCATCGAGCAAAAACTTCTGAATAATGGAAGAAGGAG
**Patch-P12h-20nt**	CCTCTCACCCACCATTCATCCTAAAGCAAGATAGAACCCTTCTGAATCGTCT
**Patch-Q01h-20nt**	CCTCTCACCCACCATTCATCGGAAAGCGACCAGGCGGATAAGTGAATAGGTG
**Patch-Q03h-20nt**	CCTCTCACCCACCATTCATCACGCAAAGGTCACCAATGAAACCAATCAAGTT
**Patch-Q05h-20nt**	CCTCTCACCCACCATTCATCGAGGCGTTAGAGAATAACATAAAAGAACACCC
**Patch-Q07h-20nt**	CCTCTCACCCACCATTCATCTTTTAGTTTTTCGAGCCAGTAATAAATTCTGT
**Patch-Q09h-20nt**	CCTCTCACCCACCATTCATCTGGATTATGAAGATGATGAAACAAAATTTCAT
**Patch-Q11h-20nt**	CCTCTCACCCACCATTCATCGCCAACAGTCACCTTGCTGAACCTGTTGGCAA
**Probe-Ala5-20nt**	(N-term → C-term) GATGAATGGTGGGTGAGAGG-(Ala) (Ala) (Ala) (Ala) (Ala)
**Probe-Thr5-20nt**	(N-term → C-term) GATGAATGGTGGGTGAGAGG-(Thr) (Thr) (Thr) (Thr) (Thr)

Table S5. P-values for the cell viability data.

**1 day**	DMSO 10%	6HB	12HB	NP	Ala	Thr	Ala_{48}-NP	Thr_{48}-NP	Ala_{96}-NP	Thr_{96}-NP
DMSO 10%		0.0222	0.00970	0.326	0.000246	0.00355	0.425	0.565	0.751	0.0264
6HB	0.0222		0.689	0.0297	0.00261	0.0218	0.0380	0.0299	0.0228	0.00564
12HB	0.00970	0.689		0.0120	0.00264	0.0258	0.0167	0.0166	0.0108	0.00286
NP	0.326	0.0297	0.0120		0.000241	0.00396	0.949	0.306	0.292	0.00900
Ala	0.000246	0.00261	0.00264	0.000241		0.410	0.000319	0.000523	0.000296	0.000167
Thr	0.00355	0.0218	0.0258	0.00396	0.410		0.00436	0.00412	0.00359	0.00196
Ala_{48}-NP	0.425	0.0380	0.0167	0.949	0.000319	0.00436		0.327	0.351	0.0167
Thr_{48}-NP	0.565	0.0299	0.0166	0.306	0.000523	0.00412	0.327		0.730	0.204
Ala_{96}-NP	0.751	0.0228	0.0108	0.292	0.000296	0.00359	0.351	0.730		0.0577
Thr_{96}-NP	0.0264	0.00564	0.00286	0.00900	0.000167	0.00196	0.0167	0.204	0.0577	

**1 week**	DMSO 10%	6HB	12HB	NP	Ala	Thr	Ala_{48}-NP	Thr_{48}-NP	Ala_{96}-NP	Thr_{96}-NP
DMSO 10%		0.0807	0.122	0.495	0.00212	0.000964	0.669	0.121	0.226	0.0520
6HB	0.0807		0.932	0.0546	0.0274	0.0266	0.0548	0.0623	0.0284	0.0137
12HB	0.122	0.932		0.0801	0.0331	0.0348	0.0859	0.0878	0.0468	0.0231
NP	0.495	0.0546	0.0801		0.00225	0.00131	0.712	0.610	0.736	0.223
Ala	0.00212	0.0274	0.0331	0.00225		0.575	0.00158	0.000638	0.00102	0.00068
Thr	0.000964	0.0266	0.0348	0.00131	0.575		0.00063	0.000124	0.00035	0.000229
Ala_{48}-NP	0.669	0.0548	0.0859	0.712	0.00158	0.00063		0.203	0.369	0.0733
Thr_{48}-NP	0.121	0.0623	0.0878	0.610	0.000638	0.000124	0.203		0.827	0.196
Ala_{96}-NP	0.226	0.0284	0.0468	0.736	0.00102	0.00035	0.369	0.827		0.206
Thr_{96}-NP	0.0520	0.0137	0.0231	0.223	0.00068	0.000229	0.0733	0.196	0.206	

**1 month**	DMSO 10%	6HB	12HB	NP	Ala	Thr	Ala_{48}-NP	Thr_{48}-NP	Ala_{96}-NP	Thr_{96}-NP
DMSO 10%		0.00980	0.00481	0.324	0.00007	0.000882	0.943	0.000525	0.00531	0.000107
6HB	0.00980		0.680	0.0306	0.0455	0.286	0.0281	0.0131	0.0108	0.00792
12HB	0.00481	0.680		0.0368	0.000377	0.0370	0.0508	1.40E-07	8.72E-07	2.71E-07
NP	0.324	0.0306	0.0368		0.000816	0.00442	0.553	0.0227	0.0161	0.00951
Ala	0.00007	0.0455	0.000377	0.000816		0.0894	0.00186	0.000003	0.000002	0.000002
Thr	0.000882	0.286	0.0370	0.00442	0.0894		0.00639	0.00009	0.00312	0.000045
Ala_{48}-NP	0.943	0.0281	0.0508	0.553	0.00186	0.00639		0.0675	0.0447	0.0283
Thr_{48}-NP	0.000525	0.0131	1.40E-07	0.0227	0.000003	0.00009	0.0675		0.000303	0.000152
Ala_{96}-NP	0.00531	0.0108	8.72E-07	0.0161	0.000002	0.00312	0.0447	0.000303		0.00119
Thr_{96}-NP	0.000107	0.00792	2.71E-07	0.00951	0.000002	0.000045	0.0283	0.000152	0.00119	
